# Drivers of gut microbiome variation within and between groups of a wild Malagasy primate

**DOI:** 10.1186/s40168-021-01223-6

**Published:** 2022-02-09

**Authors:** Katja Rudolph, Dominik Schneider, Claudia Fichtel, Rolf Daniel, Michael Heistermann, Peter M. Kappeler

**Affiliations:** 1 Behavioral Ecology & Sociobiology Unit, German Primate Center, Leibniz Institute for Primate Research, Kellnerweg 4, 37077 Göttingen, Germany; 2 Department of Sociobiology/Anthropology, Johann-Friedrich-Blumenbach Institute of Zoology and Anthropology, Georg-August University Göttingen, Kellnerweg 6, 37077 Göttingen, Germany; 3 Leibniz Science Campus “Primate Cognition”, Göttingen, Germany; 4 Genomic and Applied Microbiology and Göttingen Genomics Laboratory, Institute of Microbiology and Genetics, Georg-August University Göttingen, Grisebachstraße 8, 37077 Göttingen, Germany; 5 Endocrinology Laboratory, German Primate Center, Leibniz Institute for Primate Research, Kellnerweg 4, 37077 Göttingen, Germany

**Keywords:** *Propithecus verreauxi*, Gut microbiome, Sociality, Age, Seasonality, Sex, Dominance, Reproduction, Relatedness, Ecology

## Abstract

**Background:**

Various aspects of sociality can benefit individuals’ health. The host social environment and its relative contributions to the host-microbiome relationship have emerged as key topics in microbial research. Yet, understanding the mechanisms that lead to structural variation in the social microbiome, the collective microbial metacommunity of an animal’s social network, remains difficult since multiple processes operate simultaneously within and among animal social networks. Here, we examined the potential drivers of the convergence of the gut microbiome on multiple scales among and within seven neighbouring groups of wild Verreaux’s sifakas (*Propithecus verreauxi*) — a folivorous primate of Madagascar.

**Results:**

Over four field seasons, we collected 519 faecal samples of 41 animals and determined gut communities via 16S and 18S rRNA gene amplicon analyses. First, we examined whether group members share more similar gut microbiota and if diet, home range overlap, or habitat similarity drive between-group variation in gut communities, accounting for seasonality. Next, we examined within-group variation in gut microbiota by examining the potential effects of social contact rates, male rank, and maternal relatedness. To explore the host intrinsic effects on the gut community structure, we investigated age, sex, faecal glucocorticoid metabolites, and female reproductive state. We found that group members share more similar gut microbiota and differ in alpha diversity, while none of the environmental predictors explained the patterns of between-group variation. Maternal relatedness played an important role in within-group microbial homogeneity and may also explain why adult group members shared the least similar gut microbiota. Also, dominant males differed in their bacterial composition from their group mates, which might be driven by rank-related differences in physiology and scent-marking behaviours. Links to sex, female reproductive state, or faecal glucocorticoid metabolites were not detected.

**Conclusions:**

Environmental factors define the general set-up of population-specific gut microbiota, but intrinsic and social factors have a stronger impact on gut microbiome variation in this primate species.

Video abstract

**Supplementary Information:**

The online version contains supplementary material available at 10.1186/s40168-021-01223-6.

## Background

The abundant and diverse microbial communities that live in and on humans as well as animals are key for their hosts’ physiology, ecology, and evolution. The gut microbiota include many types of bacteria and are among the most investigated microbial communities that, *inter alia*, are essential for host metabolism [1, 2], mediate its immune system [3, 4], and even impact its behaviour [5]. Thus, the interest in understanding socio-ecological drivers of the composition of gut microbiota is widespread.

It has been argued that the acquisition of bacteria that are beneficial to the host may have contributed to the evolution of sociality [6–8]. This link arises because various aspects of sociality can benefit individuals’ health via gut bacteria transmitted through the host social environment. For example, bacteria transmitted during social interactions can enhance pathogen resistance and stimulate host immunity [9, 10], and commensal microbes might outcompete pathogens for resources or produce by-products that inhibit them altogether [6, 11]. Frequent social transmission may increase microbial diversity over time, which has been associated with improved health [12]. Thus, the host social environment and its relative contributions to the host-microbiome relationship have emerged as key topics in microbial research.

Group-living animals often share more similar gut microbial communities with group members than with outsiders [13–16]. Increased physical contact between group members facilitates the transmission of microorganisms and is therefore a prevalent mechanism for shaping distinct group microbiomes [13, 17–22]. Indirect transmissions through shared environments might also contribute to these patterns [18, 23, 24].

Still, there is variation in the composition of gut bacteria among group mates [25–27]. Explaining this variation remains difficult due to the bidirectional and dynamic host-microbiome relationship as well as the numerous factors that contribute to structuring gut communities [28–31]. In fact, the host’s diet [32–38], genetic variation [7, 39–41], and host interactions with the environment [34, 42, 43] have been found to influence the assemblage of gut microbiota. Additionally, several other factors, like age [44–47], sex [48, 49], or reproductive stage [50–53], can have structuring effects.

Metacommunity theory provides a promising evolutionary framework for exploring the dynamics of host-microbiome-relationships [27, 54, 55]. Metacommunities are defined as assemblages of multiple species (e.g. microbial communities) that live in a specified place (e.g. a host or an ecological niche), that interact with one another, and that are linked to each other through dispersal (e.g. between hosts or islands) [27, 54]. In contrast to classical community ecology, metacommunity theory posits that not only local but also regional processes affect community compositions [56, 57]. In addition, the concept of the “social microbiome” — the collective microbial metacommunity of an animal’s social network — has been introduced recently [20]. According to this concept, multiple processes operate simultaneously within and among animal social networks, thereby affecting microbial dispersal opportunities and ultimately shaping individual gut microbiomes. To better understand these processes and their consequences for gut communities, it was proposed to explore intrinsic, social, and environmental drivers of microbial composition at multiple scales, from individual and group to population and interspecific levels [20].

Here, we aimed to identify the intrinsic and extrinsic drivers of the social microbiome convergence in a wild population of Verreaux’s sifakas (*Propithecus verreauxi*) at multiple scales, i.e. the individual, within-group, and between-group level [20]. We combine one of the largest gut microbiome datasets (*n* = 519 samples) on wild lemurs with a comprehensive set of behavioural, genetic, and phenological data. During four field trips across two consecutive years, we repeatedly sampled up to 41 individuals from seven neighbouring groups for which detailed demographic data are available [58]. Verreaux’s sifakas are diurnal, frugi-folivorous primates endemic to Madagascar [58, 59]. They live in multi-male multi-female groups, with group sizes ranging between 2 and 12 individuals in our study population [58]. Previous field studies of Verreaux’s sifakas revealed that sifaka groups maintain distinct gut communities [60–62]. However, these studies addressed only some factors contributing to gut microbiome similarities within groups, had small sample sizes, and/or lacked data on key environmental variables, which could mask or alter important factors influencing the social microbiome. In order to obtain a more comprehensive understanding of the interplay among multiple intrinsic and extrinsic factors shaping gut microbiome composition, we initiated this longitudinal population study. In further contrast to previous studies, we conducted indicator species analyses and generated association networks to identify bacterial taxa associated with specific social groups [63, 64]. We also used this approach to detect unique DNA sequences (amplicon sequence variants, ASVs) associated with variation in age, social status, and reproductive state.

On the between-group scale, we examined the longitudinal dynamics in microbial structures across the whole study population, and we investigated the environmental factors that might increase similarities in the gut microbiota among groups, such as home range overlap, diet, and habitat similarities. Sifaka home ranges are stable across years and partially overlap with those of neighbouring groups, but include core areas of exclusive use [65, 66]. Direct contact between the groups is very rare, even during intergroup encounters [65, 67], making horizontal transmission via social interactions of gut bacteria less likely. However, scent-marking and over-marking, i.e. placing a mark directly on top of the mark of a conspecific, occur frequently when neighbouring groups meet [68]. Thus, shared environments and scent-marking might constitute indirect social transmission routes [68], but in a different sifaka population, the effects of home range overlap and diet on gut microbial similarities between groups were not correlated [62]. However, only the home range overlap of four groups was estimated, and only about half of the population’s dietary patterns were recorded in that study [62].

We also expanded on prior studies by applying 18S rRNA gene-based analyses to examine and compare the consumed food plants genetically. Moreover, we included measures of habitat features (e.g. tree species diversity) to estimate similarities between habitats of adjacent groups. We predicted that groups inhabiting more similar habitats, feeding on more similar diets, and sharing larger parts of their home ranges should also harbour more similar gut microbiota.

On the within-group level, we aimed to identify social drivers of microbial convergence among group members; specifically social interactions, male rank, and maternal relatedness. While sifakas devote generally little time to social activities [69], group members still engage in regular grooming bouts and use social thermoregulation, i.e. they rest in body contact with conspecifics, especially during cold nights [70, 71]. Furthermore, as other strepsirrhine primates, sifakas groom each other orally rather than manually, which may facilitate bacterial transmission between individuals. We therefore predicted that group members spending more time affiliating share more similar gut microbial communities with each other.

Dispersal in Verreaux’s sifakas is male-biassed and groups contain one clearly dominant male [72, 73]. Males generally groom more often than females, but there is no difference in grooming behaviour between dominant and subordinate males [74]. However, dominant males scent- and over-mark at significantly higher rates than females and subordinate males [75], and they have distinct hormone patterns [70, 76]. Since host physiological processes can interact with the bacterial gut microbiota [77, 78], and increased scent-marking could provide more opportunities for horizontal microbial transmission, we therefore predicted that dominant males differ in their gut communities from all other group members.

Host genetic constitution potentially affects microbial colonisation patterns, resulting in higher gut microbiota similarity among kin [79–84]. However, also direct maternal transmission to offspring in utero ([83]; but see [84]) or during delivery [85, 86], or physical contacts in the contexts of maternal care [7, 87] could drive these patterns. While most primate studies found no strong indications for kinship effects on bacterial gut microbiomes [18, 44, 61, 88], a recent large-scale study in baboons found that individuals inherited the majority of their gut communities from their ancestors [84]. Here, we predicted that maternal relatives living in the same or in different groups share more similar microbiota.

Finally, on the individual level, we investigated if and how various intrinsic factors influence individual microbial diversity and composition, and thus gut community variation among group members. The majority of previous studies in different taxa, including Verreaux’s sifakas, found no or only weak effects of age and sex [18, 44, 60, 62, 89]. Therefore, we predicted to find similar patterns. Moreover, only a few studies of wild animal populations investigated potential links between physiological variables and microbiota variation [51, 52, 90]. Since female reproductive state and male dominance status in Verreaux’s sifakas are positively correlated with faecal glucocorticoid metabolite concentrations (fGCMs) [70], we explored potential links between female reproductive states, male rank, and individual fGCMs on gut microbial diversity.

## Methods

### Study site and subjects

**Fig. 1:**
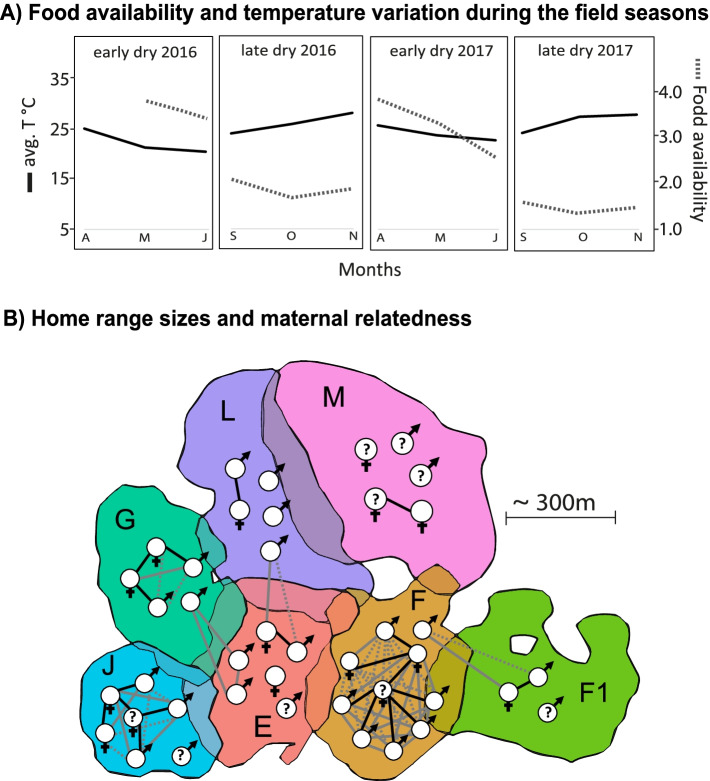
Environmental conditions, home ranges, and maternal relatedness of the study population. **A** Monthly average temperatures and food availability scores. **B** Average home range locations and overlaps of all study groups. Areas indicate the average 95% Kernels over the complete study period. Within groups’ home ranges, white circles represent individual group members and their respective sex. For individuals illustrated with question marks, we do not know the respective mothers. The degree of maternal relatedness between all individuals is indicated by connecting lines. Black solid lines: relatedness coefficient (RC) = 0.50; grey solid line: RC = 0.25–0.50; grey dotted line: RC = 0.25

This study was carried out during four field seasons (April to May 2016/2017 and September to October 2016/2017) in Kirindy Forest, western Madagascar (44° 39′ E, 20° 03′ S). The forest is a dry deciduous forest with pronounced seasonality, including a short hot, wet season (November to March), when food availability is high, and a longer, cooler dry season (April to October) when food availability is low (Fig. 1A) [58]. We observed 41 individuals belonging to seven social groups that are habituated to human’s presence and individually marked with unique collars. One group (M) only entered the study area by the end of 2016, so that data for this group were only available for the study year 2017.

### Home range dissimilarities and food availability

To characterise the habitat structure of the different home ranges, we conducted forest inventories of 10 randomly selected square plots (~ 25 × 25 m; 6 plots for group F1) within the home range of each group in 2012 [91] and 2016 [92]. We identified all trees with diameters at breast height larger than 5 cm, resulting in a data set comprising 12,177 trees of 168 different species found in 66 phenology plots (for details, see [92]). We used the number of trees per species within a groups’ home range as a proxy for evaluating habitat dissimilarity between groups (see below). Estimates of food availability for the study area were based on monthly phenology scores for leaves, fruit, and flowers of 690 trees, which ranged from 0 (complete absence) to 4 (maximum abundance) (for details, see [91]).

### Home range overlap

In a previous study [92], we assed home range sizes of each group over the same four field seasons with data collected by GPS collars. On average, we recorded GPS data points for 651 days with 21,393 ± 3,119 GPS locations per group (mean ± SD; range 17,179–24,070). For estimating home range sizes, we used monthly 95% fixed kernels with the *adehabitatHR* package [93] in Rstudio (R Version 3.6.1, [94]). Home range overlap among groups per field season was calculated with the function *kerneloverlaphr* of the *adehabitatHR* package. Figure 1B illustrates groups’ average home ranges across the study period.

### Behavioural observations

Between April 2016 and March 2018, we conducted focal animal sampling on all individuals except infants younger than 9 months. Observations lasted 1 h per individual and were conducted for 3 h in the morning and 3 h in the afternoon in a randomised but counter-balanced order. We continuously recorded social behaviours (i.e. allogrooming, play, body contact, proximity of < 1 m, and aggression), including the identity of involved conspecifics, and non-social behaviours, like feeding, locomoting, resting, and auto-grooming. In total, we collected 1812 h of behavioural data with 44 h ± 12 h per individual (mean ± SD; range 25–56 h). We additionally recorded the identity of feeding plants and parts. As our study required focal animal observations, it was not possible to record data blindly.

### Faecal glucocorticoid metabolite (fGCM) analysis

Faecal glucocorticoid metabolite (fGCM) concentrations were assessed non-invasively from faecal samples. During the four field seasons, we collected and analysed 1152 samples (3.45 ± 1.1 (mean ± SD) per animal per month). After extraction at the field site, we determined fGCM concentrations with a validated enzyme immunoassay (EIA) for the measurement of immunoreactive 11ß-hydrodroxyetiochoolanolone in the endocrinology lab of the German Primate Center. For more details on data collection and analysis, see Additional file 1 and [70].

### Bacterial and eukaryotic bacterial gut microbiome analyses

We collected and analysed 519 faecal samples during four field seasons (12.7 ± 3.6 (mean ± SD) total samples per animal; 3.9 ± 0.7 (mean ± SD) samples per animal per field season). Samples were only collected when they could be assigned to an individual. We stored samples in 2 ml polypropylene tubes containing 1 ml RNA*later* (Thermo Fisher Scientific, Waltham, MA, USA) at ambient temperature for 24h. Afterwards, samples were stored at − 20°C and shipped to Germany for further analyses.

#### Extraction of DNA, amplification, and sequencing of 16S and 18S rRNA genes

We conducted DNA extraction with the PowerSoil DNA isolation kit (MoBio, Carlsbad, Canada). PCR reactions to generate bacterial 16S rRNA gene amplicons were performed in triplicates for each sample, then pooled in equimolar amounts and cleaned. Afterwards, we conducted dual-indexed paired-end sequencing with the Illumina MiSeq platform and v3 chemistry (see Additional file 1 for details on protocols).

#### Bioinformatic processing of 16S rRNA gene amplicon sequences

Amplicon sequence variants (ASVs) were generated with VSEARCH version 2.15. We removed chimeric sequences with VSEARCH using UCHIME3 in de novo (--uchime3_denovo) and reference (--uchime_ref) mode against the SILVA SSU NR database (v138.1) [95]. We taxonomically classified ASVs with BLAST 2.9.0+ [96] against the SILVA SSU v138.1 database and removed chloroplasts and extrinsic domains from the data set. We used the following identity thresholds for taxonomical classification: species (≥ 98.7%), genus (≥ 94.5%), family (≥ 86.5%), order (≥ 82%), class (≥ 78.5%), and phylum (≥ 75%) [97]. Assignments with lower identities were marked as unclassified at the given taxonomic rank.

#### Gut community analyses

The following analyses were conducted in Rstudio (R Version 3.6.1). First, we normalised all sequences with geometric means of pairwise ratios (GMPR) (v0.1.3) [98]—a method we chose due to its robustness for zero-inflated sequencing data. To generate a phylogenetic tree all sequences of the filtered dataset were aligned with a maximum of 100 iterations using MAFFT [99]. The tree was calculated with FastTree 2.1.7 (OpeMP) [100] and then midpoint rooted and saved in newick format with FigTree (version 1.4.4) [101]. Alpha diversity was calculated with Faith’s phylogenetic diversities (PD) [102] with the *picante* package (v1.8.2). For beta diversity, we computed generalised UniFrac distances (GuniFrac) by utilising the *GuniFrac* function of the *GuniFrac* package (version 1.1) [103]. Relative abundances in the form of bar charts were generated with ggplot2 (version 3.3.3) using standard R packages. Heatmaps were built with the *ampvis2* package (version 2.6.7) [104].

#### Indicator species analysis and association networks

To identify ASVs that are significantly associated with certain “groups”, i.e. social groups, age classes, female reproductive stages, or rank, we conducted indicator species analyses using the package *indicspecies* (version 1.7.9) with the *multipatt* function [63, 64]. Therefore, samples were rarefied in *ampvis2* to 11,895 reads, based on the lower threshold for the number of reads in a sample in rarefaction curves. We calculated *point biserial correlation coefficient* with the *r.g* function and set the significance cut-off to *p* < 0.05. Cytoscape (version 3.8.2) was used to visualise association networks, using the *edge-weighted spring embedded layout* algorithm, whereby “groups” were source nodes, ASVs were target nodes, and edges weighted positive associations between ASVs and source nodes.

#### Bioinformatic processing of 18S rRNA gene amplicon sequences

ASVs were generated as described above for 16S rRNA genes. Quality-filtered sequences were mapped to chimera-free ASVs and an ASV table was created with VSEARCH. Finally, we taxonomically classified ASVs with BLASTn against the SILVA SSU NR database.

For more details on the gut microbiome analyses, see Electronic Supplementary Material (Additional file 1).

### Statistical analyses

All statistical analyses were conducted in R (versions 3.6.1 and 4.1.1).

#### Mantel tests—Beta diversity and group membership

We examined the relationship between group membership and beta diversity with Mantel tests [105] using 1000 permutations, including the original data as one permutation. Unlike PERMANOVAs, the test restricts permutations such that non-independence due to repeated sampling of the same individuals is accounted for. We conducted four Mantel tests, each including only samples of one of the four field trips. The Mantel test was restricted such that samples selected from the same individual were always permuted as a block. The test statistic yielded the mean absolute differences in dissimilarities within and between the groups. We determined *p*-values as the proportion of permutations that resulted in larger test statistics than or equal to the test statistics of the original data. The unpublished functions for this analysis were kindly provided by Dr Roger Mundry.

#### Mantel tests—Beta diversity and home range dissimilarities among groups

We examined whether groups with ecologically more similar home ranges share more similar gut microbiomes. To estimate the differences in home ranges, we computed Bray-Curtis dissimilarities among groups (calculated with the *vegdist* function of the *vegan* package) based on tree species abundances within each group’s home range. Next, we averaged GuniFrac distances for each group dyad per field season. We then calculated the Mantel tests based on Pearson’s product-moment correlation, examining the link between dyadic GuniFrac distances and dyadic habitat dissimilarity between groups for each field season.

#### LMMS

To investigate potential factors modulating Verreaux’s sifakas’ gut microbial composition, we computed linear mixed models (LMM) [106] using the function *lmer* of the *lme4* package (version 1.1.26) [107] with the optimiser “bobyqa”. For all models, covariates were *z*-transformed (transformed to a mean of zero and a SD of one) to achieve easier interpretable models [108] and to facilitate model convergence. If data on predictors were missing, e.g. age, we excluded the sample from the model. We included random slopes to keep type I error rates at the nominal level of 5% [109].

After fitting each lmer model, we controlled for assumptions of normal distributions, homoscedasticity, and collinearity, and we checked for model stability. *p*-values for individual effects were based on likelihood ratio tests comparing the full with the respective null or reduced models (R function ANOVA with argument test set to “Chisq”) [110, 111] using the *drop1* function [109]. Null models contained only intercepts, random effects, and random slopes and reduced models additionally contained assigned control factors. We obtained effect sizes of the full models for the entirety of fixed and random effects with the function *r.squaredGLMM* of the package *MuMIn* (version 1.43.17) [112]. Confidence intervals were assessed with parametric bootstrapping using an adjusted *bootMer* function from the *lme4* package. Dr. Roger Mundry also kindly provided this adjusted function.

We fitted the following models (for an overview of all models, see Tables S1 & S2).

##### LMM I—Beta diversity within the same individual

We investigated whether samples from the same individual were more similar than samples from different individuals. We included only dyads of which both individuals were from the same group. The mean GuniFrac distances per ID dyad and per field season were used as a response, the factor “Same ID” (yes or no) was used as a predictor, individual dyads and group ID were used as random effects, and field season was used as a control factor and as a random slope.

##### LMM II—Beta diversity and home range overlaps and diet dissimilarity between groups

In this model, we investigated whether groups with overlapping home ranges and groups with more similar diets share more similar gut microbiomes. The mean GuniFrac distances per group dyad per field season were used as a response, the mean home range overlaps per field season and the mean diet dissimilarity per field season were used as predictors, group dyad was used as a random effect, and field season was used as a random slope. To control for seasonal variation in ranging patterns (see [92]) and diet, we included field season as a control factor. We also compared fruit and leave intake rates between the groups and seasons in additional LMMs described in Additional file 1 (Tables S3 & S4; Fig. S1).

##### LMM III—Beta diversity and maternal relatedness

We investigated the potential effect of maternal relatedness on gut microbiome similarity among individuals. Maternal relatedness of older individuals was determined via genetic analyses in a prior study (1995–2005 [72]). For younger individuals, we used behavioural observations of mother-offspring dyads to determine relatedness [58]. We considered animals as maternally related if they were known to have one of the following degrees of kinship: RC = 0.50: mother-offspring and siblings; RC = 0.25: half-siblings, grandmother-grandchild, and aunt/uncle-nephew/niece. In case we could not determine whether individuals were full- or half-siblings, we assigned an RC of 0.375. Figure 1B illustrates all known degrees of maternal relatedness within the study population. The mean GuniFrac distances of individual dyads per field season were used as a response, the relatedness coefficient between these individuals and the interaction between relatedness coefficient and group membership (same or different) were used as predictors, individual dyad as a random effect, and field season as a control factor and random slope.

##### LMM IV—Beta diversity and intrinsic factors, affiliation, and seasonality within groups

We examined potential correlations of microbiome similarity between group members and age, sex, seasonality, and time spent affiliating, including grooming and other activities in body contact (i.e. feeding, resting). The mean GuniFrac distances between group members per field season were used as a response, and age class dyad (e.g. adult-infant), sex dyad (e.g. female-female), field season, and time spent affiliating (in min/h) were predictors. We included maternal relatedness as a control factor. Individual dyads and group ID were used as random effects and field seasons, maternal relatedness, and time spent affiliating as random slopes.

##### LMM V—Beta diversity, male rank, and group residence time

To examine the potential effects of male rank on the microbiome similarity of adult group members, we divided the rank into three categories: dominant males, subordinate adult males, and adult females. Since female dyads consisted mainly of mother-daughter pairs, we did not discern dominance relationships among them. The mean GuniFrac distances between group members per field season were used as a response; rank dyad (e. g. dominant male—adult female) and residence time, i.e. number of years two group members spent together in the same group (range 0.21–14.2, mean ± SD 5.60 ± 3.45), were predictors, and field season was included as a control factor. Individual dyads and group ID were used as random effects and field seasons as a control factor and a random slope.

##### LMM VI–IX—Alpha diversity

We applied four LMMs to examine the correlations of Faith’s phylogenetic diversity (PD) with field season, various intrinsic factors, affiliation, male rank, group ID, and diet. Models had to be separated to avoid issues with collinearity (e.g. between age and rank). In model VI, field season, sex, age, mean monthly measures of fGCMs, group ID, and mean proportions of consumed leaves per field season were predictors, animal, and group ID (except for model VI) were random effects and field season, leave intake and fGCM measures were used as random slopes. Additionally, we included age as a random slope within ID. Fruit intake rates were correlated and collinear to field season (Table S4) and therefore excluded from the model.

In model VII, we examined the effects of time spent affiliating with group members and alpha diversity. We used the mean time spent affiliating per field season as predictor, field season as a control factor, group and animal ID as random effects, and affiliation and field season as random slopes.

In model VIII, we explored the potential correlations of male rank with individual PD. Male rank was included as predictor, field season as control factor and random slope, and group and animal ID were random effects.

In model IX, we examined the correlations of female reproductive state with alpha diversity. Adult females were retrospectively categorised as “reproducing” or “not reproducing”, depending on whether they gave birth in the respective year or not. Female reproductive state and the interaction between reproductive state and season were included in the model as predictors. The interaction was included to examine differences between gestating (in the early dry season) and lactating (in the late dry season) females. Field season was included as a control factor, and random slope, group, and animal ID were random effects. Additionally, we included female reproductive state as a random slope within the group.

More details on the statistical analyses are provided in Additional file 1.

## Results

**Fig. 2:**
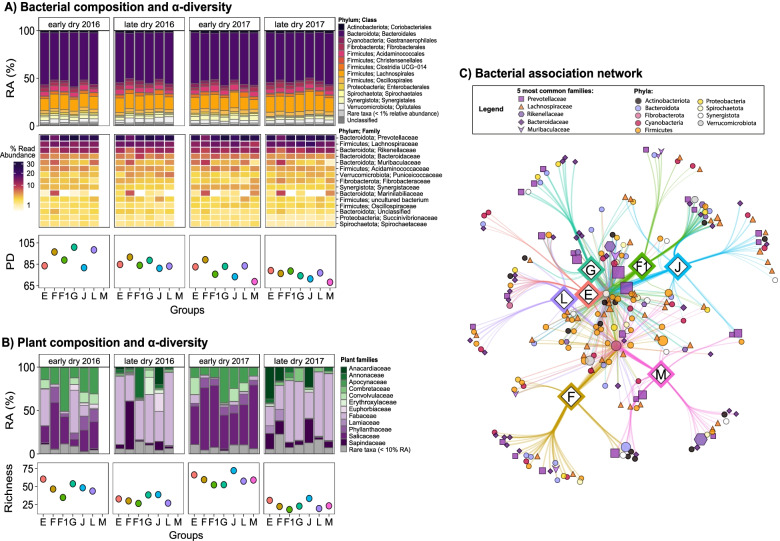
Overview of the between-group variation in the gut composition and diversity and the potentially influencing ecological and intrinsic factors. **A** Stacked barplot and heatmap of the average relative abundances of bacterial phyla and class or phyla and families, respectively, and average alpha diversity per group and field season. **B** Stacked barplot of the average relative abundances and average richness of land plant families per group and field season. **C** ASVs associated with the different groups in 2017. The graph does not contain data on 2016 since group M only joined the study population in 2017, and there were no prior data available. The association network was calculated with the *indicspecies* package in R and visualised in Cytoscape with an *edge-weighted spring embedded* layout. Branch lengths indicate the *point biserial correlation coefficient*. Each circle or other shape indicates a bacterial ASV associated (*p* < 0.05) with the group it is connected to. Coloured circles indicate phyla except for the 5 most abundant families, which are indicated by different shapes. Sizes of the circles and other shapes indicate the average relative abundance of each ASV among all samples

The 519 samples of the 16S rRNA gene-based analysis, contained 22,914,114 high-quality amplicon sequences (44,151 ± 19,080 reads (mean ± SD) per sample, range 11,941–192,639) and 6013 bacterial ASVs. A total of 90% of the ASVs could be taxonomically assigned at the phylum, class, and order level, while only 76%, 8%, and 0.4% of ASVs could be classified at the family, genus, and species level, respectively. Twelve phyla were identified in the samples: *Bacteroidota* (3179 ASVs), *Firmicutes* (1462 ASVs), *Actinobacteria* (159 ASVs), *Proteobacteria* (151 ASVs), *Cyanobacteria* (123 ASVs), *Spirochaetes* (118 ASVs), *Synergistota* (118 ASVs), *Verrucomicrobia* (57 ASVs), *Fibrobacteres* (34 ASVs), *Desulfobacterota* (25 ASVs), *Campylobacterota* (3 ASVs), and *Armatimonadota* (1 ASV). About 60% of all reads belonged to the five most common families: *Prevotellaceae* (23%), *Lachnospiraceae* (15%), *Rikenellaceae* (7%), *Bacteroidaceae* (6%), and *Muribaculaceae* (5%) (Fig. 2A).

We also conducted an 18S rRNA gene-based analysis to identify food plants contained in the faecal samples. The 519 samples contained 24,736,841 high-quality amplicon sequences (47,662 ± 32,579 reads (mean ± SD) per sample, range 2–199,773) within 6465 ASVs. Of those ASVs, 562 belonged to *Tracheophytes* (land plants) and contained 9,975,587 reads. After subsampling, 95 samples were removed due to low read numbers for *Tracheophytes* (< 1000 reads). The remaining 424 faecal samples of 41 different individuals contained 553 ASVs and 9,938,628 high-quality amplicon sequences. We identified 71 plant families. The five families with the highest numbers of ASVs were *Apocynaceae* (84), *Solanaceae* (58), *Fabaceae* (56), *Ebenaceae* (34), and *Convolvulaceae* (32). About 86% of all reads belonged to the five families *Salicaceae* (40%), *Apocynaceae* (17%), *Fabaceae* (14%), *Phyllanthaceae* (9%), and *Convolvulaceae* (6%) (Fig. 2B).

### Beta diversity: host and group membership

Samples from the same individuals were more similar than samples from different individuals of the same group (LMM I; likelihood ratio test comparing full and null/reduced model *χ*^2^ = 25.376, df = 1, *p* < 0.001, *R*^2^_m/c_ = 0.14/0.89) (Table S5). Group membership impacted the gut microbiome as samples of group members for each field season were more similar to each other than to samples from individuals living in different groups (Mantel test: early dry 2016: *n*_samples_ = 92, *n*_individuals_ = 29, $\overline{x}$_same group_ = 0.171, $\overline{x}$_different group_ = 0.228, *p* < 0.001; late dry 2016: *n*_samples_ = 116, *n*_individuals_ = 29, $\overline{x}$_same group_ = 0.172, $\overline{x}$_different group_ = 0.225, *p* < 0.001; early dry 2017: *n*_samples_ = 155, *n*_individuals_ = 39, $\overline{x}$_same group_ = 0.166, $\overline{x}$_different group_ = 0.228, *p* < 0.001; late dry 2017: *n*_samples_ = 156, *n*_individuals_ = 36, $\overline{x}$_same group_ = 0.155, $\overline{x}$_different group_ = 0.220, *p* < 0.001; Table S6). However, visual inspection of Fig. 2A indicated that all groups have very similar gut compositions up to the family level, which is why larger differences between groups must appear at the genus level or beyond.

Indicator species analysis at the ASV level revealed that only 1.33% of all taxa (i.e. 80 ASVs) were uniquely associated with one social group (Table S7). In more detail, groups seemed to differ to a larger degree within taxa of the phyla *Bacterioidota* (42 ASVs) and *Firmicutes* (17 ASVs) and their most common families *Bacteroidaceae* (19 ASVs), *Prevotellaceae* (11 ASVs), and *Lachnospiraceae* (12 ASVs). Members of groups F and M differed most strongly in their microbiome compositions compared to the other groups (Fig. 2C).

### Beta diversity: habitat dissimilarity, habitat overlap, and diet

Habitat dissimilarity and GuniFrac distances between the groups were not correlated (Mantel test: *n*_samples_ = 15, *n*_groups_ = 6, *r* = − 0.149, *p* = 0.553; late dry 2016: *n*_samples_ = 15, *n*_groups_ = 6, *r* = 0.008, *p* = 0.972; early dry 2017: *n*_samples_ = 21, *n*_groups_ = 7, *r* = − 0.154, *p* = 0.561; late dry 2017: *n*_samples_ = 21, *n*_groups_ = 7, *r* = 0.064, *p* = 0.776; Table S8). The model examining the effects of habitat overlap and diet dissimilarities on groups’ GuniFrac distances was also not significant (LMM II: *χ*^2^ = 3.264, df = 2, *p* = 0.196, *R*^2^_m/c_ = 0.08/0.98) (Table S9).

The 18S rRNA gene analysis of the land plants found in faecal samples revealed that at least at the lower taxonomic levels, i.e. until the family level, diet did not seem to affect between-group variation in microbiome composition. Despite obvious between-group variation in food plant compositions, groups’ bacterial microbiome compositions did not reflect these differences when visually inspecting the respective graphs (Fig. 2A, B). We found, however, seasonal dietary patterns. During the early dry seasons in both study years, faecal samples contained a large proportion of plants from the families *Combretaceae* and *Salicaceae*, whereas during the late dry season *Fabaceae* and *Sapindaceae* were consumed in greater amounts (Fig. 2B).

### Beta diversity: maternal relatedness

**Fig. 3:**
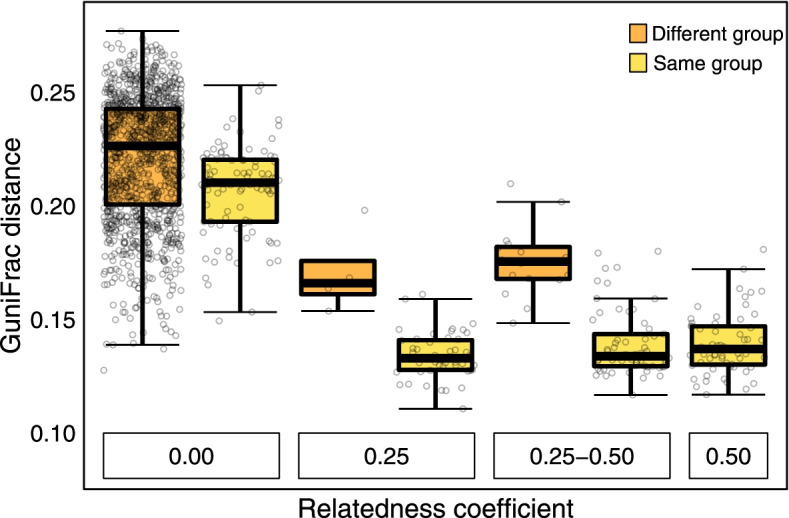
GuniFrac distances of all study animals in relation to their maternal relatedness coefficient and group membership. An RC of 0.25–0.50 refers to dyads for which we cannot determine whether they are full- or half-siblings

We examined the effects of maternal relatedness coefficients on GuniFrac distances among all individuals, i.e. between both, group members and individuals from different groups. The interaction between the relatedness coefficient and group membership (same or different) was not significant (likelihood ratio test comparing the model with and without the interaction: *χ*^2^ = 0.105, df = 1, *p* = 0.746), which is why we excluded it from the model. The model without the interaction was highly significant (LMM III:*χ*^2^ = 122.079, df = 1, *p* < 0.001, *R*^2^_m/c_ = 0.51/0.92) (Table S10). Maternal relatives had a more similar microbiome than unrelated individuals, and this effect was independent of whether these relatives lived in the same group or not (Fig. 3).

### Beta diversity: seasonality, sex, age, and affiliation rates

**Fig. 4:**
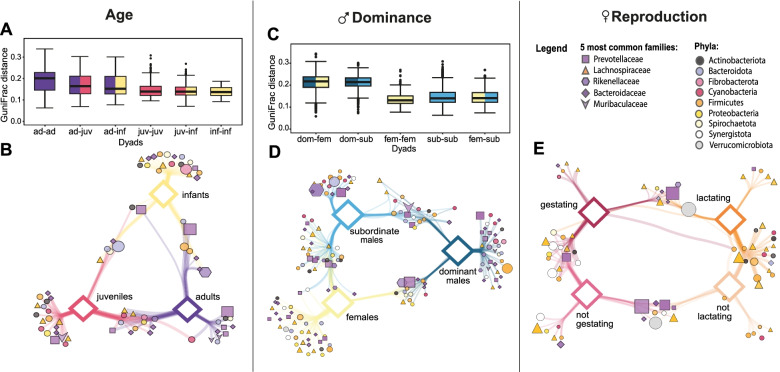
Differences in gut similarity and association networks within groups per age category, female reproductive state, and male dominance. **A**, **C** GuniFrac distances between group members of different or same age categories or rank categories of adult group members only. As there is only one dominant male per group, we could not compare two dominant individuals. We did not have enough adult female group members to compare their GuniFrac distances during different reproductive stages. **B**, **D**, **E** ASVs associated with the different age categories, adult female reproductive stages, or rank categories within groups, respectively. The association network was calculated and visualised in the same way as described in Fig. 1. The network for age categories only contains data from the late dry seasons 2016/2017 since animals were only considered infants, when they were < 9 months of age. Hence, during the early dry seasons, there were no infants in the population

The model examining correlations of dyadic GuniFrac dissimilarity with seasonality, sex, age classes, and the time two group members spent affiliating was significant (LMM IV: *χ*^*2*^ = 30.759, df = 10, p < 0.001, R^2^_m/c_ = 0.70/0.91) (Tables S11). Bacterial microbiomes of group members increased in similarity across the study period; they were least similar in the early and late dry season 2016 and most similar in the late dry season 2017. Samples of adults differed most from each other, whereas samples among juveniles and infants were more similar (Fig. 4A). Neither sex nor time spent affiliating significantly affected microbiome similarity.

Indicator species analysis on ASV level revealed that less than 1% of all taxa (i.e. 49 ASVs) were uniquely associated with one age class (Table S12). These taxa belonged mainly to the phyla *Bacterioidota* (22 ASVs) and *Firmicutes* (12 ASVs) and their respective families *Bacteroidaceae* (10 ASVs) and *Lachnospiraceae* (7 ASVs) (Fig. 4B). Only juveniles and infants had unique associations with taxa affiliated to the phylum *Firmicutes*.

### Beta diversity: male rank and female reproductive state

The model examining correlations of dyadic GuniFrac dissimilarity with male rank was significant (LMM V: *χ*^2^ = 31.827, df = 4, *p* < 0.001, *R*^2^_m/c_ = 0.68/0.92) (Table S13). Dominant males shared the least similar gut microbiota with their group members (Fig. 4C). The number of years two individuals spent together in the same group was not significantly associated with gut community similarity (Table S13).

Indicator species analysis revealed that 3.74% of all taxa, i.e. 225 ASVs, were uniquely associated with one rank category in adult Verreaux’s sifakas (Table S14). Individuals differed most strongly in taxa of the phyla *Bacterioidota* (99 ASVs) and *Firmicutes* (63 ASVs) and their most common families *Bacteroidaceae* (35 ASVs), *Prevotellaceae* (29 ASVs), and *Lachnospiraceae* (31 ASVs) (Fig. 4D).

Female reproductive stage did not appear to affect their community composition. In the indicator species analysis, we found that less than 1% of all taxa, i.e. 22 ASVs (total number of ASVs in adult female samples: 6000), were uniquely associated with one reproductive category (Table S15). Fourteen of these belonged to the phylum *Firmicutes* (Fig. 4E). Eight ASVs were associated with reproducing females (gestating and lactating) of which five belonged to *Bacterioidota* (Table S16). Non-reproducing females (not gestating and not lactating) shared 9 ASVs of which five belonged to *Bacterioidota* (Table S16).

### Alpha diversity (PD): seasonality, sex, age, group ID, fGCMs, and leave intake rates

The model examining correlations between PD and seasonality, sex, age, group ID, mean monthly fGCM concentrations, and monthly leave intake rates was significant (LMM VI: *χ*^2^ = 99.857, df = 13, *p* < 0.001, *R*^2^_m/c_ = 0.31/0.38) (Table S17). PD decreased continuously throughout the field seasons. Group membership was correlated to PD with members of group F having the highest and members of group M having the lowest diversities (Fig. 2A). Yet, group size had no influence on between-group variation in PD. For example, while the largest group (F, mean_group size_ = 10) harboured the highest diversity, the second largest group (J, mean_group size_ = 7) was on the lower end, and the smallest group (F1, mean_group size_ = 3) ranged in the middle. Sex, age, mean monthly fGCMs, and monthly leave intake rates were not correlated to PD.

### Alpha diversity: affiliation, male rank, and female reproductive state

The models examining the correlations between PD and affiliation, male rank, and female reproductive state were not significant (affiliation: LMM VII: *χ*^2^ = 1.897, df = 1, *p* = 0.168, *R*^2^_m/c_ = 0.10/0.30; male rank: LMM VIII: *χ*^2^ = 3.013, df = 2, *p* = 0.222, *R*^2^_m/c_ = 0.22/0.37; female reproductive state: LMM IX: *χ*^2^ = 0.861, df = 4, *p* = 0.930, *R*^2^_m/c_ = 0.18/0.35) (Tables S18, S19, and S20).

## Discussion

We examined social, environmental, and intrinsic drivers of between- and within-group variation in the gut microbiome of seven adjacent wild Verreaux’s sifaka groups on multiple scales. Throughout the 2-year-study period, bacterial diversity decreased for the whole study population, whereas microbial similarities between individuals and groups increased. However, none of our environmental predictors explained the causes for this population-wide pattern. As expected, throughout all field seasons group members shared more similar gut microbiota and groups differed in bacterial diversity. Between groups, neither home range overlap nor sharing of more similar habitats or diets explained variation in gut microbiome communities. Maternally related individuals shared more similar gut microbiota, both within and between groups. Interestingly, gut communities appeared to be even more similar in maternal relatives living in different groups than in maternally unrelated group members. Dominant males had significantly different gut microbiota compared to their group mates, independent of their residence time in the group. In contrast, variation in social interactions between group members was not correlated with microbial similarity. On the individual scale, despite groups sharing distinct gut communities, animals still harboured unique gut microbiota. Most variation between individuals occurred among adults and maternally unrelated group members but was independent of sex. Bacterial diversity was not correlated to any other factors but group and season.

Within each of the two study years, alpha diversity was smaller during the late than during the early dry season. In line with higher food availability and consumption of fibre-rich fruits [113], Verreaux’s sifakas’ intake in non-structural carbohydrates and fibres was highest during the early dry season [91]. Both macronutrients drive microbial diversity [114–116], explaining our findings that alpha diversity was higher in the early dry season. However, this seasonal effect contrasts with the results of a previous study of the same population, where alpha diversity was larger at the end of the dry season [60]. Either different methods and alpha diversity metrics or seasonal or annual variation in nutrients [113, 117–119] may have led to these different results.

While the here found relationship between season and bacterial diversity seems conclusive, we cannot rule out that this result is not a consequence of the observed continuous decrease in alpha and beta diversity between individuals over the course of the study period. In fact, alpha diversity was lower during the early dry season in 2017 than during the late dry season 2016. In a different population, such a population-level shift of the social microbiome was detected as well [62]. Interannual variation in hormone concentrations have been reported in Verreaux’s sifakas [70], but also in other species, like Florida scrub jays (*Aphelocoma coerulescens*), degus (*Octodon degus*), or snowshoe hares (*Lepus americanus*) [120–122], and might be linked to environmental factors. Yet, there were no major differences in temperatures or food availability between the study years [70], and neither fruit or leaf intake rates nor 18S rRNA gene deduced plant species richness were correlated to alpha diversity. Thus, while we cannot pin down the causes responsible for the continuous decline of alpha diversity, the observed pattern indicates that adjacent sifaka groups might share a mutual “micro-environment” that affects the gut microbiome of the whole population in a similar manner [62].

Kirindy Forest is very heterogenous in terms of forest structure and composition [58, 123], and sifakas’ home ranges differ in both feeding tree richness and abundance [92]. Yet, in contrast to our prediction, neither home range overlap nor similarity in habitat or diet reflected microbial similarities among groups. Instead, several other studies reported links between variation in habitat type and microbiome composition or diversity [14, 31, 32, 36, 124, 125], but they compared the groups or populations at much larger spatial scales. The comparatively minor local variation in habitat features and food supplies among sifaka groups does not seem to predict between-group variation in microbiome composition.

Our findings also imply that the horizontal transmission of bacteria via scent-marking or sharing environments is an unlikely driving force for between-group variation in this species. Verreaux’s sifakas’ arboreal lifestyle has been suggested to act as a buffer against environmentally transmitted microorganisms [126]. Physical contact with faecal material or faecal-contaminated soil likely promotes a between-group exchange of gut bacteria [12, 127], however, in arboreal species like sifaka this transmission route is less likely. Additionally, the intensity of microbial transmissions can depend on bacteria’s viability under external environmental conditions [12, 128]. Identifying and understanding the routes and conditions of these transmissions will help to understand the dynamics within the social microbiome, but for now, we lack the groundwork [129, 130].

Intriguingly, we found that maternally related individuals shared more similar gut microbiota between and within groups, contradicting results of the same species [61] but also those of several studies in other primates [16, 18, 131, 132]. Only a recent large-scale study in yellow baboons (*Papio cynocephalus*) also reported the gut microbiota to be highly heritable and pointed out that it required multiple samples of the same individuals collected over a decade to detect microbiome heritability [84], suggesting that previous studies may have had too small sample sizes.

In our study, unrelated group members shared less similar gut communities than relatives living in different groups. It is also interesting to note that groups G, L, and E, whose members are partially related to each other (Fig. 3), also clustered in terms of microbial similarities (Fig. 2C). The between-group variation could thus also be affected by kinship. Within groups, on the other hand, genetic relatedness likely adds to, but cannot be the only driver of microbial convergence in this species since related group members still shared more similar gut communities than relatives living in different groups. Because our sample size on maternally related individuals living in different groups is small, we welcome follow-up studies to verify the here found patterns.

As expected, group membership predicted microbiome composition and diversity, confirming the results of previous studies [60, 61]. However, within-group variation in affiliation did not further predict microbial similarity, contrasting findings in chimpanzees (*Pan troglodytes*) and baboons [18, 19], but confirming findings in sooty mangabeys (*Cercocebus atys*) [133] and Verreaux’s sifakas from a different population [61]. Verreaux’s sifakas devote relatively little time to social activities [69], and grooming bouts are relatively short [74], offering little opportunity for the horizontal transmission of bacteria. Meanwhile, in their small cohesive groups, cohabitation, and proximity to all group members, e.g. when huddling during cool nights, which we obviously did not protocol, may provide more opportunities for transmitting commensal bacteria across the group and thus shaping distinctive group microbiota.

Alpha diversity was neither predicted by group size nor by the time group members spent affiliating, whereas in another Verreaux’s sifaka population a positive correlation with social interactions was found [61]. The findings of the said study are based on a snapshot perspective of a small number of gut microbial samples: In addition, they constructed grooming networks on a community level to derive social network metrics for each group, whereas we used dyadic interaction rates by accounting for group membership, which we think is a more direct measure to assess social relationships. Hence, differences in study design and statistical methods may account for the discrepancy between the two studies.

In contrast to a study of savannah baboons [49], dominant males differed significantly in their gut composition from all other group members. This effect was unrelated to the time individuals spent in the same group, ruling out social co-residency as an explanation for this effect. Dominant males have higher androgen [76] and fGCM [70] concentrations than their conspecifics, and their chests are stained from scent gland secretions, whereas subordinate males’ chests are rather clean [134, 135]. Thus, they have distinct physiological phenotypes, which might be linked to their divergent gut microbial composition. Additionally, since dominant males exhibit much higher rates of scent- and overmarking than their conspecifics [67], they are exposed to substantially more opportunities for horizontal transmissions than their group members, which may promote differential gut communities. Also, the information communicated via odour cues of their scent-marks likely differs from their conspecifics, as they convey their social status or serve mate-guarding purposes [75, 136]. In fact, stained sifakas have microbial communities in their gland secretions that differ significantly from those of unstained males [137]. According to the “fermentation hypothesis”, bacteria contribute to the production of chemical signals via anaerobic fermentation [138]. If the gut microbiome was also involved in the process of odour production, this might explain aspects of inter-individual variation of gut community structures [139]. Likewise, social groups can have specific chemical signatures [140, 141] that could be linked to groups’ distinct gut microbiota. However, whereas surface microbes have been shown to be involved in the production of odorants [140, 142–144], the role of gut microbes in olfactory communication remains to be explored in vertebrates [145].

Within-group variation in gut communities was partly due to age. More precisely, faecal samples of adult group members differed the most in their bacterial compositions, whereas comparisons between other age categories yielded lower dissimilarity scores. Indicator species analyses revealed little taxonomic variation across age classes. Due to male-biassed natal dispersal, adult sifakas have lower average degrees of relatedness than younger individuals within their groups. With maternally related individuals sharing more similar gut microbiota in this study, this kinship effect probably contributes to the age effect on within-group variation.

Age-related changes in gut microbiota in mammals occur especially during the early life stages, when the introduction of solid food marks important turning points in microbial convergence towards those of adults [46, 146, 147]. However, because our study only included weaned individuals, we presumably missed the major changes in gut communities during infant development.

Measures of individual physiological states, as the here used fGCMs or female reproductive stages, did not predict variation in inter-individual bacterial diversity or composition, confirming results of previous studies [49, 60, 89]. It has been suggested that, instead of relying on coarse physiological markers, more dynamic physiological measures of hormonal or immunological activity may offer a better understanding of host-microbiome relationships [51, 148, 149]. Future studies using such proximate physiological markers may therefore yield deeper insights.

## Conclusions

We contribute to a more comprehensive understanding of the relative importance of environmental, intrinsic, and social factors shaping the social microbiome in wild primates at multiple scales, i.e. between groups, within groups, and among individuals. Our results indicate that environmental factors define the general set-up of population-specific gut microbiota, whereas more minor differences in microhabitat features or diet among local groups do not seem to inflict significant between-group variation. Kinship promotes microbial homogeneity both between and within groups, whereas male dominance rank, which is associated with a unique physiological phenotype in this species, drives inter-individual variation. Other physiological variables linked to stress and reproduction had no effects on gut microbiome similarity. Overall, intrinsic and social factors have a stronger impact on gut microbiome variation in this primate species than environmental factors.

## Supplementary Information

**Additional file 1.** Supplementary electronic material. The document contains additional details on the conducted methods and statistical analyses, result tables for all models and analyses, and an additional analysis on variation in leave and fruit consumption between groups and field seasons.

**Additional file 2.** Datasets. The document contains metadata for each analysed faecal sample and an additional table with the values for the beta diversity comparisons including the respective metadata used for the analyses.

## Data Availability

All 16S and 18S rRNA gene sequence data used in this study are available at the NCBI Sequence Read Archive (https://www.ncbi.nlm.nih.gov/) under BioProject ID PRJNA527362. The datasets supporting the conclusions of this article are included within the article and its additional files. The RScript used for the analyses of the data is available in the OSF repository, https://osf.io/Sifaka_RScript.
