# Supplementary material for: Drivers of gut microbiome variation within and between groups of a wild Malagasy primate
Source: Microbiome. 2022 Feb 9;10:28. doi: 10.1186/s40168-021-01223-6 (PMC8827170; doi:10.1186/s40168-021-01223-6)
Supplement: Supplementary file 2 — Additional file 1. Supplementary electronic material. The document contains additional details on the conducted methods and statistical analyses, result tables for all models and analyses, and an additional analysis on variation in leave and fruit consumption between groups and field seasons. [file 40168_2021_1223_MOESM2_ESM.docx]

**SUPPLEMENTARY ELECTRONIC MATERIAL**

Article title: **Drivers of gut microbiome variation within and between groups of a wild Malagasy primate**

Journal: Microbiome

Authors: Katja Rudolph^1,2,3^, Dominik Schneider^4^, Claudia Fichtel^1,3^, Rolf Daniel^4^, Michael Heistermann^5^, Peter M. Kappeler^1,2,3^

Affiliations: ^1^ Behavioral Ecology & Sociobiology Unit, German Primate Center, Leibniz Institute for Primate Research, Kellnerweg 4, 37077 Göttingen, Germany

^2^ Dept. Sociobiology /Anthropology, Johann-Friedrich-Blumenbach Institute of Zoology and Anthropology, University Göttingen, Kellnerweg 6, 37077 Göttingen, Germany

^3^ Leibniz Science Campus “Primate Cognition”, Göttingen, Germany

^4^ Genomic and Applied Microbiology and Göttingen Genomics Laboratory, Institute of Microbiology and Genetics, University Göttingen, Grisebachstraße 8, 37077 Göttingen, Germany

^5^ Endocrinology Laboratory, German Primate Center, Leibniz Institute for Primate Research, Kellnerweg 4, 37077 Göttingen, Germany

Correspondence: Katja Rudolph

🖂 krudolp@gwdg.de

Behavioral Ecology & Sociobiology Unit

German Primate Center, Leibniz Institute for Primate Research

+49 551 2011319

**Content**

[1) Methods 2](#_Toc89474610)

[a. Bacterial microbiome analyses 2](#_Toc89474611)

[b. Eukaryotic microbiome analyses 3](#_Toc89474612)

[c. Faecal glucocorticoid metabolite (fGCM) analyses 4](#_Toc89474613)

[d. More details on statistical analyses 5](#_Toc89474614)

[e. Model overview 7](#_Toc89474615)

[2) Additional analyses - Leave and fruit intake per group and field season 9](#_Toc89474616)

[3) Results 11](#_Toc89474617)

[a. Bacterial gut microbiome composition 11](#_Toc89474618)

[Effects of ID and group membership 11](#_Toc89474619)

[Between-group variation 13](#_Toc89474620)

[Within-group variation 14](#_Toc89474621)

[b. Alpha diversity 19](#_Toc89474622)

[4) References 21](#_Toc89474623)

1. Methods
2. Bacterial microbiome analyses

*Extraction of DNA, amplification, and sequencing of 16S rRNA genes*

DNA was extracted from approximately 100 mg faecal sample using the PowerSoil DNA isolation kit (MoBio, Carlsbad, USA) following the instructions of the manufacturer with minor modifications. To ensure complete homogenization of the faecal samples, cells were mechanically disrupted with a FastPrep-24 Classic Grinder (MP Biomedicals, Santa Ana, California, USA) for 20 s at 6.5 m/s. Bacterial 16S rRNA gene amplicons were generated using the forward and reverse primers S-D-Bact-0341-b-S-17 (5′- CCTACGGGNGGCWGCAG-3′) and S-D-Bact-0785-a-A-21 (5′-GACTACHVGGGTATCTAATCC-3′) [1], including the Illumina MiSeq sequencing adaptors. PCR reaction mixtures (total volume 50 μl) contained 1 U Phusion high fidelity DNA polymerase (Biozym Scientific, Oldendorf, Germany), 2.5 μl DMSO (5%), 1 µl of forward and reverse primers (10 µM), 1 µl dNTP (10 mM), 0.2 μl MgCl_2_ (50 mM), and 25 ng of isolated DNA. Thermal cycling conditions were as follows: initial denaturation for 1 min at 98 °C, 25 cycles at 98 °C for 45 s, 55 °C for 45 s, and 72 °C for 30 s, and a final extension at 72 °C for 5 min. Negative (without template) and positive controls (with genomic *Escherichia coli* DH5α DNA) were included in all PCRs. We used agarose gel electrophoresis to verify correct amplicon size (~550 bp). PCR reactions were performed in triplicates for each sample, then pooled equimolar and purified using MagSi-NGS^PREP^ Plus (Steinbrenner, Wiesenbach, Germany) as described by the supplier. The Göttingen Genomics Laboratory determined the sequences. Nextera DNA Library Prep kit was used for indexing PCR products according to the manufacturer (Illumina, San Diego, CA, USA), followed by dual-indexed paired-end sequencing with the Illumina MiSeq platform (2 x 300 bp) and v3 chemistry as recommended by the manufacturer (Illumina). Sequences were demultiplexed and adapters clipped using CASAVA data analysis software (Illumina).

*Bioinformatic processing of 16S rRNA gene amplicon sequences*

Paired-end sequencing data from the Illumina MiSeq were quality-filtered with fastq (v0.20.0) [2] using default settings with the addition of an increased per base phred score of 20, base pair corrections by overlap (-c), as well as 5′- and 3′-end read trimming with a sliding window of 4, a mean quality of 20 and minimum sequence size of 50 bp. After quality control, the paired-end reads were merged using PEAR (v0.9.11) [3] and primers clipped using cutadapt (v2.5) [4] with default settings. Sequences were then processed using VSEARCH (v2.15.0) [5]. Processing included sorting and size-filtering of the paired reads to ≥300 bp (--sortbylength --minseqlength 300) and dereplication (--derep_fulllength). Dereplicated amplicon sequence variants (ASVs) were denoised with UNOISE3 using default settings (--cluster_unoise -minsize 8) and chimeras were removed (--uchime3_denovo). An additional reference-based chimera removal was performed (--uchime_ref) against the SILVA SSU NR database (v138.1) [6]. Quality-filtered and merged reads were mapped against the ASVs (--usearch_global–id 0.97). Finally, taxonomic classification of the ASVs was performed with BLAST 2.9.0+ [7] against the SILVA SSU v138.1 database, chloroplasts and extrinsic domain ASVs were removed from the data set. In addition, the following identity thresholds proposed by Yarza et al. (2014) for taxonomical classification of 16S rRNA genes were used to improve accuracy: species (≥ 98.7%), genus (≥ 94.5%), family (≥ 86.5%), order (≥ 82%), class (≥ 78.5%), and phylum (≥ 75%). Assignments with lower identities were marked as unclassified at the given taxonomic rank.

*Gut bacterial community analysis*

The following analyses were performed on data normalised with the GMPR (v0.1.3) [9] and conducted in R (R v4.0.3, R Core Team, 2020). To generate a phylogenetic tree all sequences of the filtered dataset were aligned with a maximum of 100 iterations using MAFFT [11]. The tree was calculated with FastTree 2.1.7 (OpeMP) [12] and then midpoint-rooted and saved in Newick format with FigTree (version 1.4.4) [13]. To assess alpha diversity, i.e. the bacterial diversity within samples, we calculated Faith’s Phylogenetic Diversities (PD) [14] with the *picante* package (v1.8.2). For examinations of beta diversity, i.e. variance of community composition among samples, we computed generalized UniFrac distances (GUniFrac).GUniFrac distances detect microbial abundances but further include information on counts and adjusted weights of phylogenetic branch lengths [15]. We computed GUniFrac distances with the *GUniFrac* function of the *GUniFrac* package (version 1.1) [15]. Relative abundances in the form of bar charts were generated with ggplot2 (version 3.3.3) using standard R packages. Heatmaps were built with the *ampvis2* package (version 2.6.7) [16].

1. Eukaryotic microbiome analyses

*Extraction of DNA, amplification, and sequencing of 18S rRNA genes*

Eukaryotic 18S rRNA gene amplicons were generated using the primers TAReuk454FWD1 and TAReukREV3 [17] harbouring the Illumina MiSeq sequencing adaptors. PCR reaction mixtures (total volume 50 μl) and contained 1 U Phusion high fidelity DNA polymerase (Biozym Scientific, Oldendorf, Germany), 2.5 μl DMSO (5%), 1 µl of forward and reverse 18S rRNA gene primers (10 µM), 1 µl dNTP (10 mM), 0.2 μl MgCl_2_ (50 mM), and 50 ng of isolated DNA. Thermal cycling conditions were as follows: initial denaturation for 1 min at 98 °C, 25 cycles at 98 °C for 45 s, 60 °C for 45 s, and 72 °C for 30 s, and a final extension at 72 °C for 5 min. Negative (without template) and positive controls (genomic DNA isolated from *Aspergillus nidulans*) were included. All PCR products were checked for appropriate size via gel electrophoresis. We performed PCR reactions in triplicates for each sample, then pooled in equimolar amounts and purified using MagSi-NGS^PREP^ Plus (amsbio, Frankfurt am Main, Germany) as described by the supplier. The Göttingen Genomics Laboratory determined the sequences. Nextera DNA Library Prep kits were used for indexing PCR products according to the manufacturer’s manual (Illumina), followed by dual-indexed paired-end sequencing with the Illumina MiSeq platform (2 x 300 bp) and v3 chemistry. Sequences were demultiplexed and adapters clipped using CASAVA data analysis software (Illumina).

*Bioinformatic processing of 18S rRNA gene amplicon sequences*

Paired-end sequences were merged using PEAR with default parameters [3]. Afterwards, we removed sequences with average quality scores below 20 and/or containing unresolved bases using trimmomatic 0.36. [18]. Additionally, cut-adapt was employed with default settings to remove reverse and forward primer sequences [4]. ASVs were generated with VSEARCH [5]. In detail, reads were sorted by length and amplicons with a read length shorter 250 bp removed, afterwards, amplicons were dereplicated and denoised utilizing the UNOISE3 algorithm of VSEARCH. Chimeric sequences were removed with VSEARCH using UCHIME3 (--uchime3_denovo) in *de novo* and reference (--uchime_ref) mode against the SILVA SSU database (v138.1) [6]. Quality-filtered sequences were mapped to chimera-free ASVs and an ASV table was created with VSEARCH. Finally, ASVs were taxonomically classified with BLASTn against the SILVA SSU (v138.1) database.

1. Faecal glucocorticoid metabolite (fGCM) analyses

We collected faecal samples (mean fresh weight = 1.078 g), uncontaminated by urine, during morning observations (7 – 11 am), within 3 min after defecation from the forest floor. Samples were only collected when they could be assigned to an individual. We did not collect samples from dependent infants. Samples were in 5 ml of 80% ethanol in water. At the field site, steroid extractions took place on the evening of the same day of sample collection. We first manually homogenized all samples for 3 minutes and subsequently vortexed for 20 sec. Then, we centrifuged samples with a manually operating centrifuge (c.f. [19,20]) and decanted ~1.5ml of the supernatant into 2ml polypropylene safe-lock tubes (Eppendorf®, Hamburg, Germany) for storage at ambient temperatures in the dark [19]. Within one to six months following sample collection, we shipped faecal extracts to the Endocrinology Laboratory, where they were stored at -20°C until fGCM analysis. With a group-specific enzyme immunoassay (EIA) for the measurement of 5ß-reduced cortisol metabolites, we measured fGCM concentrations from the faecal extracts, based on samples’ wet weights (for details see [21]). This assay has been shown to reliably assess adrenocortical activity from faecal samples (e.g. [22]) in numerous primate species of all major taxa, including Verreaux’s sifakas [23]. Moreover, HPLC analysis of faecal extracts from a male and a female following an ACTH challenge test [23], showed that in terms of the number and elution positions (i.e. characteristic) of the cortisol metabolites measured, males and females did not differ (unpublished data). Inter- and intra-assay coefficients of variations (CVs) of high- and low-quality controls were 9.0% (high, n=75) and 16.0% (low, n=75) and 7.1% (high, n=17) and 8.4% (low, n=17), respectively.

1. More details on statistical analyses

LMM II - Beta diversity and home range overlaps and diet dissimilarity between groups

We investigated whether groups with overlapping home ranges and groups with more similar diets share more similar gut microbiomes. To estimate differences in diets, we computed Bray-Curtis dissimilarities between groups based on proportions of feeding times spent on different plant species per field season. Data collection on feeding behaviour for this analysis was conducted from March to May 2017 and August to October 2016/17, resulting in a total of 280 h of feeding data. For the first field season (March to May 2016) no data on consumed plant species were recorded, which is why this analysis was only performed for the three remaining field seasons. For statistical analysis, we computed a LMM in the same manner as described above.

LMM III - Beta diversity and maternal relatedness

We investigated the potential effect of maternal relatedness on gut microbiome similarity among individuals. Maternal relatedness of older individuals was determined via genetic analyses in a prior study (1995 – 2005, [24]). For younger individuals, we used behavioural observations of mother-offspring dyads to determine relatedness [25]. For 9 out of 41 individuals, mothers could not be assigned as they were absent during genetic data collection or adult individuals immigrated into the study population after 2005. These individuals were excluded from the analysis. Figure 1 illustrates all known degrees of maternal relatedness within the study population.

LMM IV - Beta diversity and intrinsic factors, affiliation and seasonality within groups

We examined potential correlations of microbiome similarity between group members and age, sex, seasonality and time spent affiliating, including grooming and other activities in body contact (i.e. feeding, resting). Behavioural data for this analysis were collected from January to May 2017 and June to October 2016/17, i.e. covering the time of three out of four field seasons plus the 3 months prior to the respective field seasons (i.e. data for the field season April-May 2016 were not included in this analysis as there were no behavioural observations conducted from January to March 2016). During this time, we collected 1,436 h of behavioural data with 35 h ± 9 h per individual (mean ± SD; range: 19 h - 45 h) of which Verreaux’s sifakas spent on average 3.7 ± 2.8 min/h (mean ± SD) affiliating. Age classes were categorised in the following way: adults (age > 4 y), juveniles (age < 4 y) and infants (age < 1 y) [25].

1. Model overview

| **Table S1 Summary of all linear mixed models on beta diversity.** Predictors are written in bold; control factors are indicated in italic. We conducted comparisons between full and reduced models using likelihood ratio tests to examine the overall significance of the predictors within a model. Therefore, full models (indicated in the table) were compared to respective reduced models, which only contained control factors, random effects, and slopes but no predictor variables. GUniFrac distances were always averaged per individual per field season. | | | |
| --- | --- | --- | --- |
| **Model** | **Sample size** | **Research question** | **Model structure** |
| **Beta diversity** | | | |
| **I** | N_Observations_ = 455  N_ID Dyads_ = 153 N_Groups_ = 7 | Are samples of the same individual more similar than samples of different group members? | GUniFrac within and between group members ~ **Same ID (yes or no) +** Season +  (1 + early dry season 2017 + early dry season 2016 + late dry season 2017 \|\| Group ID) +  (1 + early dry season 2017 + early dry season 2016 + late dry season 2017 \|\| IDDyad) |
| **II** | N_Observations_ = 57  N_GroupDyads_ = 21  N_Groups_ = 7 | Does home range overlap and/or diet dissimilarity correlate with microbiome similarity among groups? | GUniFrac between groups ~ **Overlap rate + Diet dissimilarity (Bray-Curtis)** + Season+  (1 + early dry season 2017 + late dry season 2017 + Overlap + Diet Diss \|\| Group ID) |
| **III** | N_Observations_ = 1439  N_IDDyads_ = 502 | Do maternally related individuals share more similar microbiomes? | GUniFrac between all individuals ~ **Relatedness coeff *** Group membership (same or different) + Season+  (1 + early dry season 2017 + early dry season 2016 + late dry season 2017 \|\| IDDyad) |
| **IV** | N_Observations_ = 206  N_IDDyads_ = 89  N_Groups_ = 7 | Do seasonality, intrinsic factors and/or time spent affiliating correlate with microbiome similarity among group members? | GUniFrac between group members ~ **Season + Age dyad + Sex dyad + Affiliation (min/h)** + Relatedness coeff +  (1 + early dry season 2017 + early dry season 2016 + late dry season 2017 + Body contact \|\| GroupID) +  (1 + early dry season 2017 + early dry season 2016 + late dry season 2017 + Body contact \|\| IDDyad) |
| **V** | N_Observations_ = 84  N_ID Dyads_ = 29 N_Groups_ = 6 | Does male rank and/or time spent in the same group correlate with microbiome similarity among group members? | GUniFrac between adult group members ~ **Rank dyad + Residence time**+ Season +  (1 + early dry season 2017 + early dry season 2016 + late dry season 2017 \|\| Group ID) +  (1 + early dry season 2017 + early dry season 2016 + late dry season 2017 \|\| IDDyad) |

| **Table S2 Summary of all linear mixed models on alpha diversity.** Predictors are written in bold; control factors are indicated in italic. We conducted comparisons between full and reduced models using likelihood ratio tests to examine the overall significance of the predictors within a model. Therefore, full models (indicated in the table) were compared to respective reduced models, which only contained control factors, random effects, and slopes but no predictor variables. | | | |
| --- | --- | --- | --- |
| **Model** | **Sample size** | **Research question** | **Model structure** |
| **Alpha diversity** | | | |
| **VI** | N_Samples_ = 455  N_IDs_ = 36  N_Groups_ = 7 | Do seasonality, intrinsic factors, group ID and/or leave intake rates correlate with phylogenetic diversity? | PD ~ **Season + Age + Sex + fGCs + Group ID + Leave intake rate +**  (1 + early dry season 2017 + early dry season 2016 + late dry season 2017 + age + fGCs + Leave intake rate \|\| ID) |
| **VII** | N_Samples_ = 427  N_IDs_ = 41  N_Groups_ = 7 | Does time spent affiliating with group members affect phylogenetic diversity? | PD ~ **Affiliation (m/h)** + Season  (1 + early dry season 2017 + late dry season 2017 + affiliation \|\| Group) +  (1 + early dry season 2017 + late dry season 2017 + affiliation \|\| ID) |
| **VIII** | N_Samples_ = 319  N_IDs_ = 25  N_Groups_ = 7 | Does male rank affect phylogenetic diversity? | PD (adults only) ~ **Rank +** Season +  (1 + early dry season 2017 + early dry season 2016 + late dry season 2017 \|\| Group) +  (1 + early dry season 2017 + early dry season 2016 + late dry season 2017 \|\| ID) |
| **IX** | N_Samples_ = 157  N_IDs_ = 11  N_Groups_ = 7 | Does female reproductive state affect phylogenetic diversity? | PD (adult females only) ~ **Reproducing female (yes or no) *** Season +  (1 + early dry season 2017 + early dry season 2016 + late dry season 2017 + Reproducing female \|\| Group) +  (1 + early dry season 2017 + early dry season 2016 + late dry season 2017\|\| ID) |

1. Additional analyses - Leave and fruit intake per group and field season

We applied two binomial models with beta error distribution structures and a logit link function using the *glmmTMB* package (version 1.0.2.1) [26] to examine variation in leave and fruit consumption between groups and field seasons. We used mean intake rates on leaves or fruits per individual per season as response variables, group ID and season as predictors and animal ID as random effect. P-values for individual effects were based on likelihood ratio tests comparing the full with the respective null models using the *drop1* function [27]. We encountered no issues when checking for model stability and overdispersion and assessed confidence intervals with the *confint* function of the *glmmTMB* package.

The model on leave intake rates was not significant (Leaves: *χ^2^* = 4.416, df =9, p = 0.882) whereas the model on fruit intake rates was (Fruits: *χ^2^* = 54.679, df =9, p > 0.001) (Tables S20 and S21). Intake rates of fruits were higher during the early dry season, when more food was available [25,28]. Groups did not differ significantly in their leave and fruit consumption (Figure SX).

| **Table S3** Variation in leave consumption between groups and seasons in Verreaux’s sifakas; results of the full model (GLMM; N_Observations_ = 133, N_Groups_ = 7, N_ID_ = 41). | | | | | | | | | | |
| --- | --- | --- | --- | --- | --- | --- | --- | --- | --- | --- |
| Term | | Est | SE | Lower CI | Upper CI | Min^a^ | Max^a^ | χ^2 b^ | *df* | *P* |
| (Intercept) | | 0.281 | 0.208 | -0.127 | 0.690 | -0.053 | 0.343 | ^c^ | ^c^ | ^c^ |
| Group^d^ | |  |  |  |  |  |  | 2.999^e^ | 6^e^ | 0.809^e^ |
|  | F | -0.308 | 0.212 | -0.725 | 0.108 | -0.398 | -0.190 | ^c^ | ^c^ | ^c^ |
|  | F1 | -0.234 | 0.293 | -0.808 | 0.341 | -0.440 | 0.083 | ^c^ | ^c^ | ^c^ |
|  | G | -0.284 | 0.246 | -0.767 | 0.199 | -0.409 | 0.026 | ^c^ | ^c^ | ^c^ |
|  | J | -0.218 | 0.232 | -0.673 | 0.238 | -0.331 | 0.071 | ^c^ | ^c^ | ^c^ |
|  | L | -0.280 | 0.254 | -0.778 | 0.218 | -0.494 | 0.027 | ^c^ | ^c^ | ^c^ |
|  | M | -0.021 | 0.301 | -0.611 | 0.569 | -0.233 | 0.333 | ^c^ | ^c^ | ^c^ |
| Season^d^ | |  |  |  |  |  |  | 1.458^e^ | 3^e^ | 0.692^e^ |
|  | late dry 2017 | -0.100 | 0.195 | -0.482 | 0.282 | -0.253 | -0.001 | ^c^ | ^c^ | ^c^ |
|  | early dry 2016 | 0.022 | 0.202 | -0.375 | 0.418 | -0.229 | 0.213 | ^c^ | ^c^ | ^c^ |
|  | early dry 2017 | 0.115 | 0.192 | -0.262 | 0.492 | 0.012 | 0.291 | ^c^ | ^c^ | ^c^ |
| ^a^ Minimum and maximum of model estimates obtained when dropping levels of random effects one at a time  ^b^ Results of a likelihood ratio test comparing the full model with a reduced model lacking the respective term  ^c^ Not shown as not having a meaningful interpretation. See footnotes of Table S1 for details.  ^d^ Manually dummy-coded with group “E” and season “late dry” being the reference categories  ^e^ Values refer to the overall test of the effect of the predictors (“Group”, “Season”), not the specific level indicated in the respective row | | | | | | | | | | |

| **Table S4** Variation in fruit consumption between groups and seasons in Verreaux’s sifakas; results of the full model (GLMM; N_Observations_ = 133, N_Groups_ = 7, N_ID_ = 41). | | | | | | | | | | |
| --- | --- | --- | --- | --- | --- | --- | --- | --- | --- | --- |
| Term | | Est | SE | Lower CI | Upper CI | Min^a^ | Max^a^ | χ^2 b^ | *df* | *P* |
| (Intercept) | | -1.710 | 0.238 | -2.175 | -1.244 | -2.091 | -1.638 | ^c^ | ^c^ | ^c^ |
| Group^d^ | |  |  |  |  |  |  | 8.279^e^ | 6^e^ | 0.218^e^ |
|  | F | -0.498 | 0.245 | -0.978 | -0.017 | -0.529 | -0.462 | ^c^ | ^c^ | ^c^ |
|  | F1 | -0.589 | 0.346 | -1.266 | 0.088 | -0.647 | -0.118 | ^c^ | ^c^ | ^c^ |
|  | G | -0.088 | 0.270 | -0.617 | 0.441 | -0.096 | 0.359 | ^c^ | ^c^ | ^c^ |
|  | J | 0.011 | 0.250 | -0.478 | 0.501 | -0.018 | 0.480 | ^c^ | ^c^ | ^c^ |
|  | L | -0.055 | 0.289 | -0.621 | 0.511 | -0.129 | 0.447 | ^c^ | ^c^ | ^c^ |
|  | M | -0.386 | 0.364 | -1.099 | 0.327 | -0.481 | 0.088 | ^c^ | ^c^ | ^c^ |
| Season^d^ | |  |  |  |  |  |  | 50.769^e^ | 3^e^ | **<0.001^e^** |
|  | late dry 2017 | -0.454 | 0.243 | -0.929 | 0.022 | -0.570 | -0.262 | ^c^ | ^c^ | ^c^ |
|  | early dry 2016 | 0.684 | 0.238 | 0.219 | 1.150 | 0.443 | 0.917 | ^c^ | ^c^ | ^c^ |
|  | early dry 2017 | 1.115 | 0.227 | 0.669 | 1.560 | 0.931 | 1.302 | ^c^ | ^c^ | ^c^ |
| ^a^ Minimum and maximum of model estimates obtained when dropping levels of random effects one at a time  ^b^ Results of a likelihood ratio test comparing the full model with a reduced model lacking the respective term  ^c^ Not shown as not having a meaningful interpretation. See footnotes of Table S1 for details.  ^d^ Manually dummy-coded with group “E” and season “late dry 2016” being the reference categories  ^e^ Values refer to the overall test of the effect of the predictors (“Group”, “Season”), not the specific level indicated in the respective row | | | | | | | | | | |


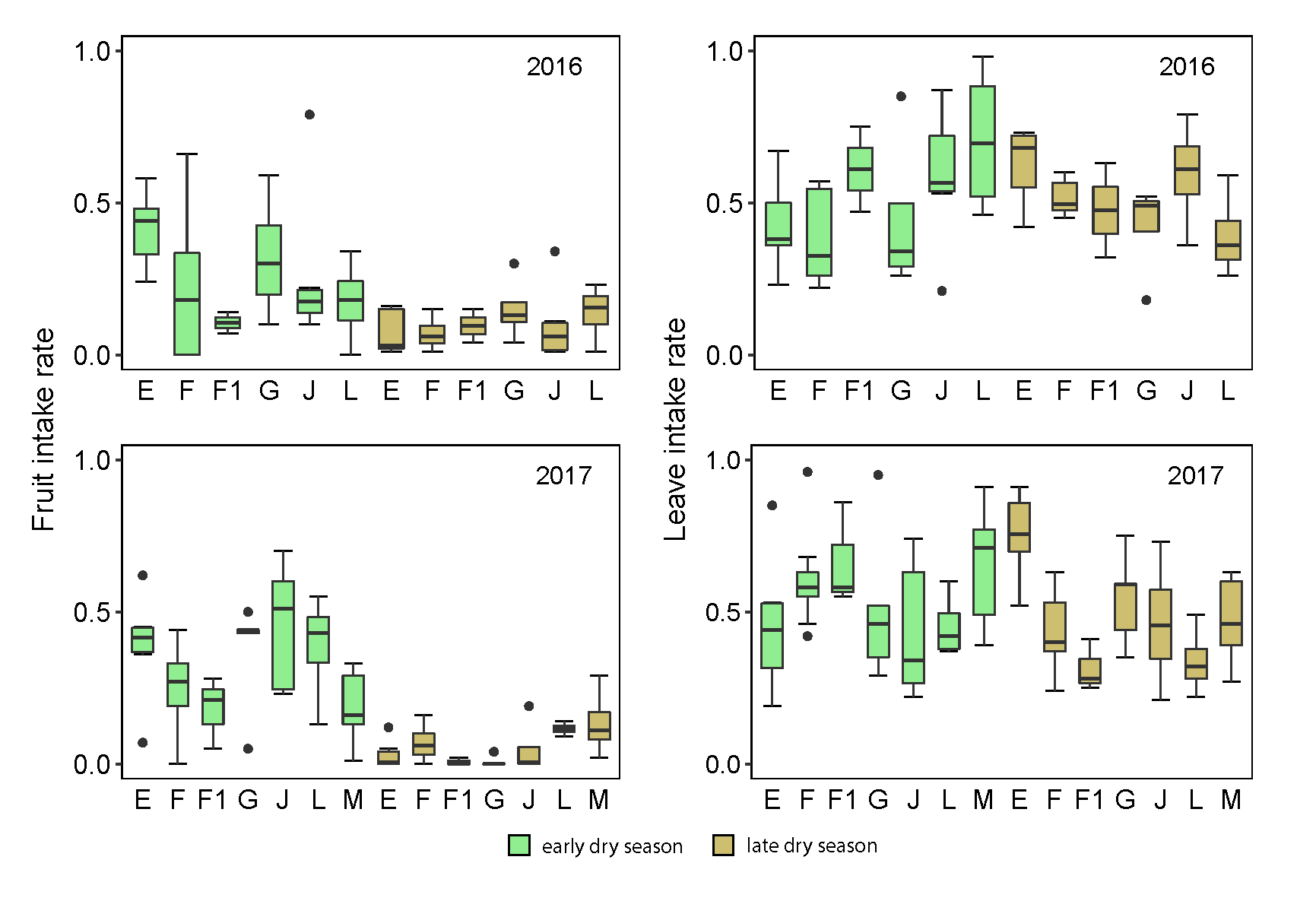


**Figure S1**: Groups’ average leave and fruit intake rates during all field seasons.

1. Results
2. Bacterial gut microbiome composition

Effects of ID and group membership

LMM I: Beta diversity within the same individual

| **Table S5** Comparing seasonally averaged GUniFrac distances within and between group members in seven groups of Verreaux’s sifakas; results of the full model (LMM; N_Observations_ = 455, N_ID dyads_ = 153). | | | | | | | | | | |
| --- | --- | --- | --- | --- | --- | --- | --- | --- | --- | --- |
| Term | | Est | SE | Lower CI | Upper CI | Min^a^ | Max^a^ | χ^2 b^ | *df* | *P* |
| (Intercept) | | 0.178 | 0.006 | 0.167 | 0.189 | 0.174 | 0.183 | ^c^ | ^c^ | ^c^ |
| Same ID (yes)^d^ | | -0.030 | 0.006 | -0.041 | -0.018 | -0.034 | -0.027 | 25.376 | 1 | **<0.001** |
| Season^d^ | |  |  |  |  |  |  | 9.213^e^ | 3^e^ | **0.027^e^** |
|  | late dry 2017 | -0.008 | 0.002 | -0.013 | -0.004 | -0.011 | -0.007 | ^c^ | ^c^ | ^c^ |
|  | early dry 2016 | 0.001 | 0.003 | -0.005 | 0.006 | -0.001 | 0.003 | ^c^ | ^c^ | ^c^ |
|  | early dry 2017 | -0.005 | 0.002 | -0.010 | -0.001 | -0.007 | -0.004 | ^c^ | ^c^ | ^c^ |
| ^a^ Minimum and maximum of model estimates obtained when dropping levels of random effects one at a time  ^b^ Results of a likelihood ratio test comparing the full model with a reduced model lacking the respective term  ^c^ Not shown as not having a meaningful interpretation. For intercepts, p-values would refer to estimated GUniFrac distances, when all covariates are at zero. For main effects of predictors which are involved in interaction terms, p-values refer only to the effect of that involved predictor with the interacting covariate at zero. This means the main effects of predictors involved in interactions depend on the value of the other main effects and are, therefore, not interpretable in themselves. Therefore, we consider p-values of main effects to be meaningful only when the predictors are not involved in an interaction.  ^d^ Manually dummy-coded with the reference categories being “Same ID (no)” and “late dry 2016”  ^e^ Values refer to the overall test of the effect of the predictor (“Season”), not the specific level indicated in the respective row | | | | | | | | | | |

Mantel tests - Beta diversity and group membership

| **Table S6** Results of Mantel tests comparing GUniFrac distances within and between seven groups of Verreaux’s sifakas. | | | | | |
| --- | --- | --- | --- | --- | --- |
| Season | n_samples_ | n_individuals_ | x̄_same group_ | x̄_different group_ | *P* |
| early dry 2016 | 92 | 29 | 0.171 | 0.228 | **<0.001** |
| late dry 2016 | 116 | 29 | 0.172 | 0.225 | **<0.001** |
| early dry 2017 | 155 | 39 | 0.166 | 0.220 | **<0.001** |
| late dry 2017 | 156 | 36 | 0.158 | 0.220 | **<0.001** |

Indicator species analysis: Social groups

| **Table S7** Overview of the number of ASVs that were significantly associated with only one group of Verreaux’s sifakas. | | | | | | | | | |
| --- | --- | --- | --- | --- | --- | --- | --- | --- | --- |
| **Phyla and families** | **E** | **F** | **F1** | **G** | **J** | **L** | **M** | **Total** |  |
| **Actinobacteriota** |  |  |  |  | **1** |  | **2** | **3** |  |
| Coriobacteriaceae |  |  |  |  |  |  | 1 | 1 |  |
| Eggerthellaceae |  |  |  |  | 1 |  |  | 1 |  |
| uncultured |  |  |  |  |  |  | 1 | 1 |  |
| **Bacteroidota** | **3** | **14** | **4** | **9** | **4** | **3** | **5** | **42** |  |
| Bacteroidaceae | 2 | 6 |  | 4 | 2 | 2 | 3 | 19 |  |
| Muribaculaceae |  | 1 | 2 |  |  |  |  | 3 |  |
| Prevotellaceae |  | 3 | 1 | 3 | 2 | 1 | 1 | 11 |  |
| Rikenellaceae |  |  |  |  |  |  | 1 | 1 |  |
| Tannerellaceae | 1 | 1 |  | 1 |  |  |  | 3 |  |
| Unclassified |  | 3 | 1 | 1 |  |  |  | 5 |  |
| **Cyanobacteria** | **2** | **3** | **2** |  | **2** | **1** |  | **10** |  |
| Candidatus |  |  |  |  | 1 |  |  | 1 |  |
| uncultured bacterium | 1 | 1 | 2 |  |  | 1 |  | 5 |  |
| uncultured rumen | 1 | 2 |  |  | 1 |  |  | 4 |  |
| **Firmicutes** |  | **4** | **2** |  | **5** | **3** | **3** | **17** |  |
| Lachnospiraceae |  | 2 | 2 |  | 5 | 1 | 2 | 12 |  |
| Eubacteriaceae |  |  |  |  |  |  | 1 | 1 |  |
| uncultured bacterium |  | 2 |  |  |  | 2 |  | 4 |  |
| **Proteobacteria** |  | **1** |  | **2** |  |  |  | **3** |  |
| Succinivibrionaceae |  | 1 |  | 2 |  |  |  | 3 |  |
| **Spirochaetota** |  | **1** |  |  |  |  | **1** | **2** |  |
| Spirochaetaceae |  | 1 |  |  |  |  | 1 | 2 |  |
| **Synergistota** |  |  |  | **1** | **1** |  | **1** | **3** |  |
| Synergistaceae |  |  |  | 1 | 1 |  | 1 | 3 |  |
| **Total** | **5** | **23** | **8** | **12** | **13** | **7** | **12** | **80** |  |

Mantel test - Beta diversity and habitat dissimilarity between groups

| **Table S8** Results of Mantel tests comparing beta diversity between seven groups of Verreaux’s sifakas (GUniFrac) with groups’ habitat dissimilarities (Bray-Curtis). | | | | |
| --- | --- | --- | --- | --- |
| Season | n_samples_ | ng_roups_ | r | *P* |
| early dry 2016 | 15 | 6 | -0.149 | 0.533 |
| late dry 2016 | 15 | 6 | 0.008 | 0.972 |
| early dry 2017 | 21 | 7 | -0.154 | 0.561 |
| late dry 2017 | 21 | 7 | 0.064 | 0.776 |

Between-group variation

LMM II - Beta diversity and home range overlaps and diet dissimilarity between groups

| **Table S9** Correlation of habitat overlap and diet dissimilarity (Bray-Curtis) on seasonally averaged GUniFrac distances among seven groups of Verreaux’s sifakas; results of the full model (LMM; N_Observations_ = 57, N_Group dyads_ = 21). | | | | | | | | | | |
| --- | --- | --- | --- | --- | --- | --- | --- | --- | --- | --- |
| Term | | Est | SE | Lower CI | Upper CI | Min^a^ | Max^a^ | χ^2 b^ | *df* | *P* |
| (Intercept) | | 0.224 | 0.004 | 0.216 | 0.230 | 0.222 | 0.226 | ^c^ | ^c^ | ^c^ |
| Habitat overlap^d^ | | 0.003 | 0.002 | -0.001 | 0.007 | 0.002 | 0.004 | 3.052 | 1 | 0.081 |
| Diet Dissimilarity^d^ | | -0.001 | 0.001 | -0.003 | 0.002 | -0.001 | 0.000 | 0.143 | 1 | 0.706 |
| Season^e^ | |  |  |  |  |  |  | 20.869^f^ | 2^f^ | **<0.001^f^** |
|  | late dry 2017 | -0.009 | 0.001 | -0.012 | -0.007 | -0.010 | -0.009 | ^c^ | ^c^ | ^c^ |
|  | early dry 2017 | -0.005 | 0.002 | -0.008 | -0.001 | -0.007 | -0.004 | ^c^ | ^c^ | ^c^ |
| ^a^ Minimum and maximum of model estimates obtained when dropping levels of random effects one at a time  ^b^ Results of a likelihood ratio test comparing the full model with a reduced model lacking the respective term  ^c^ Not shown as not having a meaningful interpretation. See footnotes of Table A.1 for details.  ^d^ z-transformed, mean and SD of the original values were 0.042 and 0.057 for overlap and 0.592 and 0.132 for diet diversity, respectively  ^e^ Manually dummy-coded with the season “late dry 2016” being the reference category  ^f^ Values refer to the overall test of the effect of the predictor (“Season”), not the specific level indicated in the respective row | | | | | | | | | | |

LMM III - Beta diversity and maternal relatedness

| **Table S10** Correlations of maternal relatedness (relatedness coefficient) with seasonally averaged GUniFrac distances in seven groups of Verreaux’s sifakas; results of the full model (LMM; N_Observations_ = 1439, N_ID dyads_ = 502). | | | | | | | | | | |
| --- | --- | --- | --- | --- | --- | --- | --- | --- | --- | --- |
| Term | | Est | SE | Lower CI | Upper CI | Min^a^ | Max^a^ | χ^2 b^ | *df* | *P* |
| (Intercept) | | 0.218 | 0.002 | 0.215 | 0.221 | 0.222 | 0.226 | ^c^ | ^c^ | ^c^ |
| Relatedness coeff.^d^ | | -0.019 | 0.002 | -0.023 | -0.016 | 0.002 | 0.003 | 122.079 | 1 | **<0.001** |
| Same group (yes)^e^ | | -0.025 | 0.004 | -0.033 | -0.016 | -0.002 | 0.000 | 32.456 | 1 | **<0.001** |
| Season^e^ | |  |  |  |  |  |  | 130.133^f^ | 3^f^ | **<0.001^f^** |
|  | late dry 2017 | -0.008 | 0.001 | -0.01 | -0.006 | -0.010 | -0.009 | ^c^ | ^c^ | ^c^ |
|  | early dry 2016 | 0.003 | 0.001 | 0.001 | 0.005 | -0.006 | -0.003 | ^c^ | ^c^ | ^c^ |
|  | early dry 2017 | -0.006 | 0.001 | -0.007 | -0.004 | 0.004 | 0.005 | ^c^ | ^c^ | ^c^ |
| ^a^ Minimum and maximum of model estimates obtained when dropping levels of random effects one at a time  ^b^ Results of a likelihood ratio test comparing the full model with a reduced model lacking the respective term  ^c^ Not shown as not having a meaningful interpretation. See footnotes of Table A.1 for details.  ^d^ z-transformed, mean and SD of the original values were 0.05 and 0.134, respectively  ^e^ Manually dummy-coded with the reference categories being “Same group (no)” and “late dry 2016” being the reference categories  ^f^ Values refer to the overall test of the effect of the predictor (“Season”) | | | | | | | | | | |

Within-group variation

LMM IV - Beta diversity and intrinsic factors, affiliation and seasonality within groups

| **Table S11** Correlations of age category, sex, time spent affiliating, and season with seasonally averaged GUniFrac distances between group members of seven groups of Verreaux’s sifakas; results of the full model (LMM; N_Observations_ = 206, N_ID dyads_ = 89, N_Groups_ =7). | | | | | | | | | | |
| --- | --- | --- | --- | --- | --- | --- | --- | --- | --- | --- |
| Term | | Est | SE | Lower CI | Upper CI | Min^a^ | Max^a^ | χ^2 b^ | *df* | *P* |
| (Intercept) | | 0.205 | 0.007 | 0.190 | 0.220 | 0.197 | 0.209 | ^c^ | ^c^ | ^c^ |
| Age category^d^ | |  |  |  |  |  |  | 22.495^f^ | 5 ^f^ | **<0.001^f^** |
|  | adult - infant | -0.015 | 0.005 | -0.026 | -0.006 | -0.018 | -0.014 | ^c^ | ^c^ | ^c^ |
|  | infant - infant | -0.004 | 0.014 | -0.066 | -0.012 | -0.044 | -0.036 | ^c^ | ^c^ | ^c^ |
|  | adult - juvenile | -0.008 | 0.004 | -0.015 | -0.001 | -0.011 | -0.006 | ^c^ | ^c^ | ^c^ |
|  | juvenile - infant | -0.028 | 0.006 | -0.04 | -0.016 | -0.033 | -0.025 | ^c^ | ^c^ | ^c^ |
|  | juvenile - juvenile | -0.019 | 0.005 | -0.028 | -0.008 | -0.023 | -0.015 | ^c^ | ^c^ | ^c^ |
| Sex dyad^d^ | |  |  |  |  |  |  | 3.64^f^ | 2 ^f^ | 0.162^f^ |
|  | female - male | 0.008 | 0.006 | -0.003 | 0.019 | 0.004 | 0.013 | ^c^ | ^c^ | ^c^ |
|  | male - male | 0.013 | 0.006 | 0.000 | 0.027 | 0.008 | 0.016 | ^c^ | ^c^ | ^c^ |
| Affiliation ^e^ | | 0.001 | 0.002 | -0.002 | 0.004 | -0.001 | 0.002 | 0.233 | 1 | 0.629 |
| Season^d^ | |  |  |  |  |  |  | 6.377^f^ | 2^f^ | **<0.041^f^** |
|  | late dry 2017 | -0.001 | 0.003 | -0.018 | -0.003 | -0.014 | -0.008 | ^c^ | ^c^ | ^c^ |
|  | early dry 2017 | -0.003 | 0.002 | -0.007 | 0.001 | -0.004 | -0.002 | ^c^ | ^c^ | ^c^ |
| Relatedness coeff.^e^ | | -0.134 | 0.010 | -0.154 | -0.111 | -0.141 | -0.114 | 87.671 | 1 | **<0.001** |
| ^a^ Minimum and maximum of model estimates obtained when dropping levels of random effects one at a time  ^b^ Results of a likelihood ratio test comparing the full model with a reduced model lacking the respective term  ^c^ Not shown as not having a meaningful interpretation. See footnotes of Table A.1 for details.  ^d^ Manually dummy-coded with the reference categories being “adult-adult”, “female-female”, and “late dry 2016”  ^e^ z-transformed, mean and SD of the original values were 0.794 and 1.256 min/h and 0.249 and 0.200, respectively  ^f^ Values refer to the overall test of the effect of the predictor (“Age category”, ”Sex dyad”, “Season”) | | | | | | | | | | |

Indicator species analysis: Age classes

| **Table S12: Overview of the number of ASVs that were significantly associated with only one age class.** | | | | |
| --- | --- | --- | --- | --- |
| **Phyla and families** | **adults** | **infants** | **juveniles** | **Total** |
| **Actinobacteriota** | **1** | **2** | **2** | **5** |
| Atopobiaceae | 0 | 0 | 1 | 1 |
| Coriobacteriaceae | 1 | 0 | 0 | 1 |
| Eggerthellaceae | 0 | 2 | 0 | 2 |
| uncultured | 0 | 0 | 1 | 1 |
| **Bacteroidota** | **9** | **6** | **7** | **22** |
| Bacteroidaceae | 2 | 4 | 4 | 10 |
| Muribaculaceae | 2 | 0 | 1 | 3 |
| Prevotellaceae | 3 | 1 | 1 | 5 |
| Tannerellaceae | 1 | 1 | 1 | 3 |
| Unclassified | 1 | 0 | 0 | 1 |
| **Cyanobacteria** | **0** | **1** | **3** | **4** |
| Candidatus | 0 | 1 | 0 | 1 |
| uncultured rumen | 0 | 0 | 3 | 3 |
| **Desulfobacterota** | **0** | **1** | **0** | **1** |
| Desulfovibrionaceae | 0 | 1 | 0 | 1 |
| **Fibrobacterota** | **0** | **1** | **0** | **1** |
| Fibrobacteraceae | 0 | 1 | 0 | 1 |
| **Firmicutes** | **0** | **6** | **6** | **12** |
| Acidaminococcaceae | 0 | 0 | 1 | 1 |
| Lachnospiraceae | 0 | 3 | 4 | 7 |
| Eubacteriaceae | 0 | 1 | 1 | 2 |
| uncultured bacterium | 0 | 2 | 0 | 2 |
| **Proteobacteria** | **0** | **0** | **1** | **1** |
| Succinivibrionaceae | 0 | 0 | 1 | 1 |
| **Spirochaetota** | **1** | **2** | **0** | **3** |
| Spirochaetaceae | 0 | 1 | 0 | 1 |
| Unclassified | 1 | 1 | 0 | 2 |
| **Total** | **11** | **19** | **19** | **49** |

LMM V – Beta diversity, male rank and group residence time

| **Table S13** Correlations of rank dyads with seasonally averaged GUniFrac distances between adults of seven groups of Verreaux’s sifakas; results of the full model (LMM; N_Observations_ = 84, N_ID dyads_ = 29, N_Groups_ =6). | | | | | | | | | | |
| --- | --- | --- | --- | --- | --- | --- | --- | --- | --- | --- |
| Term | | Est | SE | Lower CI | Upper CI | Min^a^ | Max^a^ | χ^2 b^ | *df* | *P* |
| (Intercept) | | 0.210 | 0.007 | 0.196 | 0.224 | 0.204 | 0.223 | ^c^ | ^c^ | ^c^ |
| Rank dyad^d^ | |  |  |  |  |  |  | 19.917^f^ | 4^f^ | **<0.001^f^** |
|  | Dom-Subordinate | 0.011 | 0.011 | -0.011 | 0.033 | -0.001 | 0.019 | ^c^ | ^c^ | ^c^ |
|  | Female-Female | -0.058 | 0.014 | -0.085 | -0.03 | -0.069 | -0.041 | ^c^ | ^c^ | ^c^ |
|  | Subordinate-Female | -0.036 | 0.008 | -0.053 | -0.018 | -0.066 | -0.021 | ^c^ | ^c^ | ^c^ |
|  | Subordinate-Subordinate | -0.059 | 0.019 | -0.096 | -0.022 | -0.076 | -0.048 | ^c^ | ^c^ | ^c^ |
| Year spent in same group^e^ | | -0.006 | 0.005 | -0.016 | 0.004 | -0.016 | 0.001 | 1.394 | 1 | 0.238 |
| Season^d^ | |  |  |  |  |  |  | 6.335^f^ | 3^f^ | 0.096^f^ |
|  | late dry 2017 | -0.011 | 0.006 | -0.025 | 0.001 | -0.016 | -0.005 | ^c^ | ^c^ | ^c^ |
|  | early dry 2016 | -0.008 | 0.008 | -0.024 | 0.008 | -0.012 | -0.002 | ^c^ | ^c^ | ^c^ |
|  | early dry 2017 | -0.008 | 0.003 | -0.015 | -0.002 | -0.009 | -0.007 | ^c^ | ^c^ | ^c^ |
| ^a^ Minimum and maximum of model estimates obtained when dropping levels of random effects one at a time  ^b^ Results of a likelihood ratio test comparing the full model with a reduced model lacking the respective term  ^c^ Not shown as not having a meaningful interpretation. See footnotes of Table A.1 for details.  ^d^ Manually dummy-coded with the reference categories being “Dom-Dom” and “late dry 2016”  ^e^ z-transformed, mean and SD of the original values were 5.6 years and 3.5 years, respectively  ^f^ Values refer to the overall test of the effect of the predictors (“Rank dyad”, “Season”) | | | | | | | | | | |

Indicator species analysis: Male rank

| **Table S14: Overview of the number of ASVs that were significantly associated with only one rank category.** | | | | |
| --- | --- | --- | --- | --- |
| **Phyla and families** | **dominant males** | **females** | **subordinate males** | **Total** |
| **Actinobacteriota** | **3** | **6** | **4** | **13** |
| Coriobacteriaceae | 1 | 2 | 1 | **4** |
| Eggerthellaceae | 2 | 3 | 2 | **7** |
| uncultured | 0 | 1 | 1 | **2** |
| **Bacteroidota** | **33** | **37** | **29** | **99** |
| Bacteroidaceae | 11 | 15 | 9 | **35** |
| Marinilabiliaceae | 0 | 0 | 1 | **1** |
| Muribaculaceae | 3 | 0 | 1 | **4** |
| Prevotellaceae | 9 | 11 | 9 | **29** |
| Rikenellaceae | 4 | 4 | 4 | **12** |
| Tannerellaceae | 4 | 4 | 3 | **11** |
| Unclassified | 2 | 3 | 2 | **7** |
| **Cyanobacteria** | **7** | **3** | **8** | **18** |
| gut metagenome | 0 | 0 | 1 | **1** |
| uncultured bacterium | 5 | 2 | 2 | **0** |
| uncultured organism | 1 | 0 | 1 | **2** |
| uncultured rumen | 1 | 1 | 4 | **6** |
| **Desulfobacterota** | **1** | **2** | **1** | **4** |
| Desulfovibrionaceae | 1 | 2 | 1 | **4** |
| **Fibrobacterota** | **1** | **0** | **0** | **1** |
| Fibrobacteraceae | 1 | 0 | 0 | **1** |
| **Firmicutes** | **16** | **23** | **24** | **63** |
| Acidaminococcaceae | 2 | 2 | 1 | **5** |
| Christensenellaceae | 0 | 0 | 1 | **1** |
| Eubacteriaceae | 1 | 2 | 2 | **5** |
| Lachnospiraceae | 6 | 14 | 11 | **31** |
| Oscillospiraceae | 0 | 1 | 1 | **2** |
| Ruminococcaceae | 1 | 0 | 1 | **2** |
| uncultured bacterium | 5 | 4 | 7 | **16** |
| uncultured rumen | 1 | 0 | 0 | **1** |
| **Proteobacteria** | **1** | **6** | **1** | **8** |
| Enterobacteriaceae | 0 | 2 | 0 | **2** |
| Succinivibrionaceae | 1 | 4 | 1 | **6** |
| **Spirochaetota** | **3** | **3** | **1** | **7** |
| Spirochaetaceae | 2 | 3 | 1 | **6** |
| Unclassified | 1 | 0 | 0 | **1** |
| **Synergistota** | **3** | **4** | **4** | **11** |
| Synergistaceae | 3 | 4 | 4 | **11** |
| **Verrucomicrobiota** | **0** | **1** | **0** | **1** |
| Puniceicoccaceae | 0 | 1 | 0 | **1** |
| **Total** | **68** | **85** | **72** | **225** |

Indicator species analysis: Female reproductive state

| **Table S15: Overview of the number of ASVs that were significantly associated with only one female reproductive state.** | | | | | |
| --- | --- | --- | --- | --- | --- |
| **Phyla and families** | **gestating** | **not gestating** | **lactating** | **not lactating** | **Total** |
| **Bacteroidota** | **1** | **1** | **1** | **0** | **3** |
| Bacteroidaceae | 1 | 1 | 0 | 0 | **2** |
| Prevotellaceae | 0 | 0 | 1 | 0 | **1** |
| **Cyanobacteria** | **1** | **1** | **0** | **1** | **3** |
| uncultured bacterium | 1 | 0 | 0 | 1 | **2** |
| uncultured rumen | 0 | 1 | 0 | 0 | **1** |
| **Desulfobacterota** | **0** | **1** | **0** | **0** | **1** |
| Desulfovibrionaceae | 0 | 1 | 0 | 0 | **1** |
| **Firmicutes** | **3** | **3** | **4** | **4** | **14** |
| Lachnospiraceae | 2 | 2 | 3 | 1 | **8** |
| uncultured bacterium | 1 | 1 | 1 | 3 | **6** |
| **Synergistota** | **0** | **1** | **0** | **0** | **1** |
| Synergistaceae | 0 | 1 | 0 | 0 | **1** |
| **Total** | **5** | **7** | **5** | **5** | **22** |

| **Table S16: Overview of the number of ASVs that were significantly associated with reproducing females or not reproducing females.** | | | | |
| --- | --- | --- | --- | --- |
| **Phyla and families** | **gestating and lactating** |  | **Phyla and families** | **not gestating and not lactating** |
| **Bacteroidota** | **5** |  | **Bacteroidota** | **5** |
| Bacteroidaceae | 2 |  | Bacteroidaceae | 2 |
| Prevotellaceae | 1 |  | Prevotellaceae | 3 |
| Rikenellaceae | 1 |  | **Firmicutes** | **2** |
| Unclassified | 1 |  | Lachnospiraceae | 1 |
| **Firmicutes** | **2** |  | uncultured bacterium | 1 |
| Lachnospiraceae | 2 |  | **Synergistota** | **1** |
| **Proteobacteria** | **1** |  | Synergistaceae | 1 |
| Succinivibrionaceae | 1 |  | **Verrucomicrobiota** | **1** |
|  |  |  | Puniceicoccaceae | 1 |
| **Total** | **8** |  | **Total** | **9** |

1. Alpha diversity

LMM VI – Alpha diversity, field season, Group ID, age, sex, fGCs and leave intake

| **Table S17** Correlations of group, season, age, sex, monthly mean fGCs and rates of leave intake with PDs in seven groups of Verreaux’s sifakas; results of the full model (LMM; N_Observations_ = 471, N_ID_ = 36, N_Groups_ =7). | | | | | | | | | | |
| --- | --- | --- | --- | --- | --- | --- | --- | --- | --- | --- |
| Term | | Est | SE | Lower CI | Upper CI | Min^a^ | Max^a^ | χ^2 b^ | *df* | *P* |
| (Intercept) | | 84.851 | 1.738 | 81.141 | 88.292 | 83.725 | 86.013 | c | ^c^ | ^c^ |
| Group^d^ | |  |  |  |  |  |  | 35.368^e^ | 6^e^ | **0.001^e^** |
|  | F | 5.474 | 1.566 | 2.578 | 8.737 | 4.546 | 7.005 | c | ^c^ | ^c^ |
|  | F1 | -1.482 | 2.144 | -5.618 | 3.114 | -2.880 | -0.254 | c | ^c^ | ^c^ |
|  | G | 3.085 | 1.821 | -0.466 | 6.871 | 1.926 | 4.513 | c | ^c^ | ^c^ |
|  | J | -5.952 | 1.814 | -9.339 | -2.212 | -6.980 | -4.565 | c | ^c^ | ^c^ |
|  | L | 2.135 | 1.947 | -1.309 | 6.028 | 0.852 | 3.613 | c | ^c^ | ^c^ |
|  | M | -12.498 | 3.915 | -20.014 | -4.885 | -13.866 | -10.661 | c | ^c^ | ^c^ |
| Season^d^ | |  |  |  |  |  |  | 53.565^e^ | 3^e^ | **<0.001^e^** |
|  | late dry 2017 | -10.379 | 1.42 | -13.232 | -7.400 | -10.849 | -9.708 | c | ^c^ | ^c^ |
|  | early dry 2016 | 5.119 | 1.824 | 1.437 | 8.775 | 4.033 | 5.709 | c | ^c^ | ^c^ |
|  | early dry 2017 | -3.428 | 1.52 | -6.460 | -0.380 | -3.918 | -2.695 | ^c^ | ^c^ | ^c^ |
| Age in years | | 0.663 | 0.617 | -0.521 | 1.900 | 0.238 | 0.935 | 1.114 | 1 | 0.291 |
| Sex (male) | | 0.511 | 1.202 | -2.019 | 2.971 | -0.056 | 1.145 | 0.180 | 1 | 0.671 |
| fGCs | | 0.988 | 0.573 | -0.202 | 2.090 | 0.795 | 1.306 | 2.953 | 1 | 0.086 |
| Leave intake | | 0.683 | 0.748 | -0.708 | 2.185 | 0.350 | 0.997 | 0.832 | 1 | 0.362 |
| ^a^ Minimum and maximum of model estimates obtained when dropping levels of random effects one at a time  ^b^ Results of a likelihood ratio test comparing the full model with a reduced model lacking the respective term  ^c^ Not shown as not having a meaningful interpretation. See footnotes of Table A.1 for details.  ^d^ Manually dummy-coded with with the reference categories being “Group E”, “late dry 2016” and “female”  ^e^ z-transformed, mean and SD of the original values were 7.8 and 6.6 (age in years), 0.304 and 0.131 (fGCs in µg/g) and 0.51 and 0.19 (Leave intake rates), respectively  ^f^ Values refer to the overall test of the effect of the predictors (“Group”, “Season”) | | | | | | | | | | |

LMM VII – Alpha diversity and affiliation

| **Table S18** Correlations of time spent affiliating with PDs in seven groups of Verreaux’s sifakas; results of the full model (LMM; N_Observations_ = 427, N_ID_ = 41, N_Groups_ =7). | | | | | | | | | | |
| --- | --- | --- | --- | --- | --- | --- | --- | --- | --- | --- |
| Term | | Est | SE | Lower CI | Upper CI | Min^a^ | Max^a^ | χ^2 b^ | *df* | *P* |
| (Intercept) | | 84.048 | 2.082 | 79.815 | 88.139 | 82.642 | 85.500 | ^c^ | ^c^ | ^c^ |
| Affiliation^d^ | | -1.005 | 0.703 | -2.441 | 0.327 | -1.311 | -0.581 | 1.897 | 1 | 0.168 |
| Season^e^ | |  |  |  |  |  |  | 16.080^f^ | 2^f^ | **0.001^f^** |
|  | late dry 2017 | -9.253 | 1.997 | -13.342 | -5.170 | -10.118 | -7.982 | ^c^ | ^c^ | ^c^ |
|  | early dry 2017 | -4.605 | 1.450 | -7.728 | -1.682 | -5.384 | -3.895 | ^c^ | ^c^ | ^c^ |
| ^a^ Minimum and maximum of model estimates obtained when dropping levels of random effects one at a time  ^b^ Results of a likelihood ratio test comparing the full model with a reduced model lacking the respective term  ^c^ Not shown as not having a meaningful interpretation. See footnotes of Table A.1 for details.  ^d^ z-transformed, mean and SD of the original values were 3.856 and 2.881 min/h, respectively  ^e^ Manually dummy-coded with the reference category being “late dry 2016”  ^f^ Values refer to the overall test of the effect of the predictors (“Season”) | | | | | | | | | | |

LMM VIII – Alpha diversity and male rank

| **Table S19** Correlations of rank with PDs in adult individuals from seven groups of Verreaux’s sifakas; results of the full model (LMM; N_Observations_ = 319, N_ID_ = 25, N_Groups_ =7). | | | | | | | | | | |
| --- | --- | --- | --- | --- | --- | --- | --- | --- | --- | --- |
| Term | | Est | SE | Lower CI | Upper CI | Min^a^ | Max^a^ | χ^2 b^ | *df* | *P* |
| (Intercept) | | 86.651 | 2.263 | 82.453 | 90.855 | 84.403 | 87.544 | ^c^ | ^c^ | ^c^ |
| Rank^d^ | |  |  |  |  |  |  | 3.013^e^ | 2^e^ | 0.222^e^ |
|  | female | -1.238 | 1.506 | -4.270 | 1.734 | -2.425 | 0.051 | ^c^ | ^c^ | ^c^ |
|  | subordinate | -3.533 | 1.959 | -7.512 | 0.235 | -4.718 | -2.150 | ^c^ | ^c^ | ^c^ |
| Season^d^ | |  |  |  |  |  |  | 26.144^e^ | 3^e^ | **0.001^e^** |
|  | late dry 2017 | -9.928 | 1.663 | -12.975 | -6.949 | -11.066 | -8.963 | ^c^ | ^c^ | ^c^ |
|  | early dry 2016 | 6.281 | 3.180 | 0.274 | 12.379 | 4.019 | 8.702 | ^c^ | ^c^ | ^c^ |
|  | early dry 2017 | -6.036 | 1.747 | -9.384 | -2.652 | -7.119 | -5.014 | ^c^ | ^c^ | ^c^ |
| ^a^ Minimum and maximum of model estimates obtained when dropping levels of random effects one at a time  ^b^ Results of a likelihood ratio test comparing the full model with a reduced model lacking the respective term  ^c^ Not shown as not having a meaningful interpretation. See footnotes of Table A.1 for details  ^d^ Manually dummy-coded with the reference categories being “dominant” and “late dry 2016”  ^e^ Values refer to the overall test of the effect of the predictors (“Rank” and “Season”), respectively | | | | | | | | | | |

LMM IX– Alpha diversity and female reproductive state

| **Table S20** Correlations of reproductive state with PDs in adult females from seven groups of Verreaux’s sifakas; results of the full model (LMM; N_Observations_ = 157, N_ID_ = 11, N_Groups_ =7). | | | | | | | | | | |
| --- | --- | --- | --- | --- | --- | --- | --- | --- | --- | --- |
| Term | | Est | SE | Lower CI | Upper CI | Min^a^ | Max^a^ | χ^2 b^ | *df* | *P* |
| (Intercept) | | 87.103 | 4.603 | 77.229 | 96.044 | 82.100 | 91.050 | ^c^ | ^c^ | ^c^ |
| Reproducing (yes) ^d^ | | -1.433 | 4.791 | -11.317 | 8.567 | -5.855 | 3.454 | ^c^ | ^c^ | ^c^ |
| Season^d^ | |  |  |  |  |  |  | ^c^ | ^c^ | ^c^ |
|  | late dry 2017 | -11.550 | 4.869 | -20.737 | -1.423 | -16.496 | -3.296 | ^c^ | ^c^ | ^c^ |
|  | early dry 2016 | 7.721 | 7.176 | -6.925 | 22.739 | -10.900 | 26.635 | ^c^ | ^c^ | ^c^ |
|  | early dry 2017 | -7.564 | 5.020 | -17.747 | 2.664 | -12.281 | -2.542 | ^c^ | ^c^ | ^c^ |
| Reproducing*Season | |  |  |  |  |  |  | 0.631^e^ | 3^e^ | 0.889^e^ |
|  | Repr * late dry 2017 | 1.525 | 5.982 | -11.559 | 13.252 | -6.046 | 9.233 | ^c^ | ^c^ | ^c^ |
|  | Repr * early dry 2016 | -4.255 | 7.792 | -20.368 | 12.079 | -23.665 | 13.849 | ^c^ | ^c^ | ^c^ |
|  | Repr * early dry 2017 | 1.295 | 6.128 | -10.918 | 13.726 | -3.107 | 7.093 | ^c^ | ^c^ | ^c^ |
| ^a^ Minimum and maximum of model estimates obtained when dropping levels of random effects one at a time  ^b^ Results of a likelihood ratio test comparing the full model with a reduced model lacking the respective term  ^c^ Not shown as not having a meaningful interpretation. See footnotes of Table A.1 for details  ^d^ Manually dummy-coded with the reference categories being “no” and “late dry 2016”, respectively  ^e^ Values refer to the overall test of the interaction (“Reproducing*Season”), respectively | | | | | | | | | | |

1. References

1. Klindworth A, Pruesse E, Schweer T, Peplies J, Quast C, Horn M, et al. Evaluation of general 16S ribosomal RNA gene PCR primers for classical and next-generation sequencing-based diversity studies. Nucleic Acids Res. 2013;41:e1–e1.

2. Chen S, Zhou Y, Chen Y, Gu J. fastp: An ultra-fast all-in-one FASTQ preprocessor. Bioinformatics. 2018;34:i884–90.

3. Zhang J, Kobert K, Flouri T, Stamatakis A. PEAR: a fast and accurate Illumina Paired-End reAd mergeR. Bioinformatics. 2014;30:614–20.

4. Martin M. Cutadapt removes adapter sequences from high-throughput sequencing reads. EMBnet.journal. 2011;17:10–2.

5. Edgar RC. Search and clustering orders of magnitude faster than BLAST. Bioinformatics. 2010;26:2460–1.

6. Quast C, Pruesse E, Yilmaz P, Gerken J, Schweer T, Yarza P, et al. The SILVA ribosomal RNA gene database project: improved data processing and web-based tools. Nucleic Acids Res. 2013;41:D590–6.

7. Altschul SF, Gish W, Miller W, Myers EW, Lipman DJ. Basic local alignment search tool. J Mol Biol. 1990;215:403–10.

8. Yarza P, Yilmaz P, Pruesse E, Glöckner FO, Ludwig W, Schleifer K-H, et al. Uniting the classification of cultured and uncultured bacteria and archaea using 16S rRNA gene sequences. Nat Rev Microbiol. Nature Publishing Group; 2014;12:635–45.

9. Chen L, Reeve J, Zhang L, Huang S, Wang X, Chen J. GMPR: A robust normalization method for zero-inflated count data with application to microbiome sequencing data. PeerJ. 2018;6:e4600.

10. R Core Team. R: A language and environment for statistical computing. [Internet]. Vienna, Austria: R Foundation for Statistical Computing; 2020. Available from: https://www.R-project.org/

11. Katoh K, Standley DM. MAFFT Multiple Sequence Alignment Software Version 7: Improvements in Performance and Usability. Mol Biol Evol. 2013;30:772–80.

12. Price MN, Dehal PS, Arkin AP. FastTree 2 – Approximately Maximum-Likelihood Trees for Large Alignments. PLOS ONE. Public Library of Science; 2010;5:e9490.

13. Rambaut A. FigTree - tree figure drawing tool [Internet]. University of Edingburgh: Institute of Evolutionary Biology; 2018 [cited 2021 Mar 29]. Available from: http://tree.bio.ed.ac.uk/software/figtree/

14. Faith DP. Conservation evaluation and phylogenetic diversity. Biol Conserv. 1992;61:1–10.

15. Chen J, Bittinger K, Charlson ES, Hoffmann C, Lewis J, Wu GD, et al. Associating microbiome composition with environmental covariates using generalized UniFrac distances. Bioinformatics. 2012;28:2106–13.

16. Skytte KA, Kirkegaard RH, Karst SM, Albertsen M. ampvis2: an R package to analyse and visualise 16S rRNA amplicon data. bioRxiv. 2018;

17. Stoeck T, Bass D, Nebel M, Christen R, Jones MDM, Breiner H-W, et al. Multiple marker parallel tag environmental DNA sequencing reveals a highly complex eukaryotic community in marine anoxic water. Mol Ecol. 2010;19 Suppl 1:21–31.

18. Caporaso JG, Kuczynski J, Stombaugh J, Bittinger K, Bushman FD, Costello EK, et al. QIIME allows analysis of high-throughput community sequencing data. Nat Methods. 2010;7:335–6.

19. Rimbach R, Heymann EW, Link A, Heistermann M. Validation of an enzyme immunoassay for assessing adrenocortical activity and evaluation of factors that affect levels of fecal glucocorticoid metabolites in two New World primates. Gen Comp Endocrinol. 2013;191:13–23.

20. Shutt K, Setchell JM, Heistermann M. Non-invasive monitoring of physiological stress in the Western lowland gorilla (Gorilla gorilla gorilla): Validation of a fecal glucocorticoid assay and methods for practical application in the field. Gen Comp Endocrinol. 2012;179:167–77.

21. Heistermann M, Ademmer C, Kaumanns W. Ovarian cycle and effect of social changes on adrenal and ovarian function in Pygathrix nemaeus. Int J Primatol. 2004;25:689–708.

22. Heistermann M, Palme R, Ganswindt A. Comparison of different enzymeimmunoassays for assessment of adrenocortical activity in primates based on fecal analysis. Am J Primatol. 2006;68:257–73.

23. Fichtel C, Kraus C, Ganswindt A, Heistermann M. Influence of reproductive season and rank on fecal glucocorticoid levels in free-ranging male Verreaux’s sifakas (Propithecus verreauxi). Horm Behav. 2007;51:640–8.

24. Kappeler PM, Schäffler L. The lemur syndrome unresolved: Extreme male reproductive skew in sifakas (Propithecus verreauxi), a sexually monomorphic primate with female dominance. Behav Ecol Sociobiol. 2007;62:1007–15.

25. Kappeler PM, Fichtel C. A 15-year perspective on the social organization and life history of sifaka in Kirindy forest. In: Kappeler PM, Watts DP, editors. Long-Term Field Stud Primates [Internet]. Springer Berlin; 2012 [cited 2016 Mar 3]. p. 101–21. Available from: http://link.springer.com/chapter/10.1007/978-3-642-22514-7_5

26. Brooks ME, Kristensen K, van Benthem KJ, Magnusson A, Berg CW, Nielsen A, et al. glmmTMB balances speed and flexibility among packages for zero-inflated generalized linear mixed modeling. R J. 2017;9:378–400.

27. Barr DJ, Levy R, Scheepers C, Tily HJ. Random effects structure for confirmatory hypothesis testing: Keep it maximal. J Mem Lang. 2013;68:255–78.

28. Rudolph K, Fichtel C, Schneider D, Heistermann M, Koch F, Daniel R, et al. One size fits all? Relationships among group size, health, and ecology indicate a lack of an optimal group size in a wild lemur population. Behav Ecol Sociobiol. 2019;73:132.
